# Gut Failure as a Potential Unmeasured Modifier of Micronutrient Response in Critical Illness: A Structured Narrative Review of Absorption, Metabolism, and Immune Function

**DOI:** 10.3390/nu18213516

**Published:** 2026-10-26

**Authors:** Stefano Marelli, Alessandro Girombelli, Paolo Formenti, Francesco Vetrone

**Affiliations:** 1 Neuro Critical Care Unit, ASST Grande Ospedale Metropolitano Niguarda, 20162 Milan, Italy; 2 Department of Anesthesiology and Critical Care Medicine, Ospedale SS. Annunziata, 12038 Savigliano, Italy; alessandro.girombelli91@gmail.com; 3 SC Anestesia, Rianimazione e Terapia del Dolore, ASST Nord Milano, Via Gorki 50, 20092 Cinisello Balsamo, Italy; formenti.paolo80@gmail.com; 4 Pain Medicine and Intensive Care Unit, Department of Anesthesiology, Policlinico San Pietro, Ponte San Pietro, 24036 Bergamo, Italy; f.vetrone.md@gmail.com

**Keywords:** micronutrients, critical illness, gut failure, acute gastrointestinal injury, dysbiosis, gut barrier, immunonutrition, intestinal permeability, ICU, microbiome

## Abstract

Background: Micronutrient abnormalities are frequent in critically ill patients and are associated with organ dysfunction, infection, and adverse outcomes. Yet randomized controlled trials of supplementation have largely failed to demonstrate a consistent mortality or organ-failure benefit. One possible contributor to this translational gap is a systematically under-characterized variable: gut failure. In critical illness, the intestinal barrier is disrupted, epithelial cell mass is depleted, transporter expression is impaired, the intestinal microvasculature exhibits severe dysfunction, and the microbiome undergoes profound dysbiosis. These alterations may collectively impair the absorptive, metabolic, and immunological functions upon which micronutrient homeostasis depends. Purpose: This structured narrative review (SANRA-guided; 202 records) first outlines the epidemiology of micronutrient deficiencies in the ICU and defines gut failure as a biological entity distinct from ileus. It then traces the mechanistic pathways by which a dysfunctional gut disrupts key micronutrients and links this gut–micronutrient axis to immune dysregulation. Overview: We critically appraise landmark ICU supplementation trials and examine whether the absence of gastrointestinal phenotyping, reliance on circulating surrogates of bioavailability, and neglect of microbiome–nutrient interactions may have contributed to heterogeneous or negative findings. Finally, we propose a hypothesis-generating, gut-integrated framework for future studies, comprising gut integrity stratification, microbiome phenotyping, phase-adapted timing and route selection, and microbiome restoration, to realign micronutrient interventions with the biology of the gut–microbiome–immune axis. Direct evidence that gut dysfunction modifies the response to supplementation is lacking.

## 1. Introduction

Micronutrient deficiencies are common in critically ill patients [1,2]. However, low circulating concentrations do not necessarily indicate true deficiency, as they may also reflect inflammation, redistribution, capillary leak, altered binding proteins and acute-phase responses. Despite strong biological plausibility, consistent epidemiological associations, randomized controlled trials (RCTs) of micronutrient supplementation have generally produced limited or inconsistent clinical benefit [3,4,5].

Mainstream interpretations have focused on pre-existing deficits, extracorporeal losses, and inflammatory redistribution, sometimes downplaying the intestine as a major site of micronutrient absorption, metabolism, and immune regulation [6,7,8,9]. The intestinal epithelium, its vascularization, and resident microbial community are targets of critical illness, yet they are often disregarded during clinical trial design and clinical stratification. Gut failure in the ICU encompasses a spectrum of epithelial, vascular, absorptive, and microbial abnormalities, including disruption of epithelial tight junctions [10], villous atrophy, transporter downregulation [11], enterocyte apoptosis, microvascular/endothelial dysfunction, and dysbiosis [12,13,14,15,16]. Impaired splanchnic perfusion, intestinal edema, inflammation, and reduced luminal nutrient exposure may further compromise mucosal integrity and absorptive capacity [11,12,13,17,18]. These alterations may compromise micronutrient absorption and intestinal immune function. Accordingly, supplementation with vitamins and minerals may be insufficient when severe intestinal dysfunction, wasting, or malabsorption remains unrecognized or unaddressed.

Critically ill patients with shock undergo acute loss of enterocyte mass, reflected by plasma citrulline and elevated intestinal fatty acid-binding protein (I-FABP), which independently predict 28-day mortality and correlate with gastrointestinal dysfunction severity [19,20]. Progressive enteral delivery of vitamins and trace elements fails to normalize plasma levels during the first ICU week, with no relationship between intake and circulating concentrations [4], which is compatible with, but does not prove, a contribution of malabsorption, immune activation, and capillary leak to the limited effect of supplementation, particularly for the enteral route [21]. Intravenous (IV) administration may provide more predictable systemic exposure when enteral absorption is uncertain. However, bypassing intestinal absorption does not necessarily ensure tissue uptake, intracellular utilization, or restoration of immune function [22,23]. The biological response to supplementation therefore depends on a continuum extending from delivery and absorption to systemic distribution, tissue utilization, and immune-cell function.

Understanding vitamins and minerals as regulators of immune homeostasis presupposes a tissue capable of receiving, metabolizing, and translating that signal into immunological action, a role fulfilled principally by the gut-associated lymphoid tissue and the intestinal epithelium. The inflammatory state causes a profound shift in the distribution of micronutrients, along with increased consumption by the immune system itself [24]. When gut failure develops, the molecular pathways linking micronutrient metabolism and homeostasis with immune competence may be disrupted. Together, these processes may amplify systemic inflammation and multiple organ dysfunction [25], ultimately impairing the intestine’s absorptive and immunological function [12,13,26]. Advanced gastrointestinal dysfunction may therefore reduce the predictability of micronutrient delivery and biological response [4,19,20,21,22,23,27].

The scale of the problem justifies a closer look at this question. Sepsis alone accounted for an estimated 166 million (95% uncertainty interval 135–201) cases and 21.4 million (20.3–22.5) all-cause sepsis-related deaths globally, representing 31.5% of total global deaths in 2021 [28]. Among critically ill adults, a meta-analysis of 14 studies reported a prevalence of acute gastrointestinal injury (AGI) of 40% (95% confidence interval [CI] 27–54%), with higher mortality in patients with AGI than in those without (risk ratio 2.01, 95% CI 1.20–3.37) [29]; in a prospective multicenter cohort of 550 patients, 470 (85%) had some degree of AGI during the first ICU week [30]. Low vitamin D status, one of the best-studied micronutrient abnormalities, was associated with infection, sepsis, and in-hospital mortality across 14 observational reports involving 9715 critically ill patients [31]. Despite these associations, supplementation has not translated into consistent benefit: in a meta-analysis of 34 randomized trials (4678 patients), the apparent reduction in mortality with antioxidant micronutrients was not confirmed in trials at low risk of bias (relative risk 1.08, 95% CI 0.95–1.23) [5], and the six landmark ICU trials summarized in Section 3.5 randomized approximately 4300 patients without demonstrating a consistent clinical benefit, with a signal of harm for high-dose intravenous vitamin C. Understanding why associations and interventions diverge is therefore a clinically relevant, high-burden question, and the gut is a plausible but rarely measured part of the answer.

This review therefore examines gut failure as a plausible modifier of micronutrient absorption, metabolism, and immune response in critical illness and explores how gastrointestinal phenotyping could be incorporated into future mechanistic and interventional studies.

Previous reviews have addressed micronutrient therapy in the critically ill [1,3,32,33] and gut failure as a driver of critical illness [13,34] largely separately. The specific contribution of this review is threefold: (i) it brings these two fields of literature together in a testable effect-modification framework; (ii) it distinguishes, for each mechanism, evidence demonstrated in critically ill patients from evidence derived from human studies outside the ICU and from animal or in vitro models (Section 2.3, Table 1, and Supplementary Table S1); and (iii) it translates the framework into hypothesis-driven recommendations for trial design and, provisionally, into practical implications for bedside care (Section 3.7). We emphasize from the outset that gut failure is considered here as a plausible but currently insufficiently tested modifier of the response to micronutrient supplementation, not as an established explanation for the negative trials.

## 2. Materials and Methods

### 2.1. Search Strategy

This article is a structured narrative review, not a systematic review. Its reporting was guided by the six items of the Scale for the Assessment of Narrative Review Articles (SANRA) [35], and the selection process is documented using the flow-diagram format of the PRISMA 2020 statement [36] for transparency only, as the review does not claim to meet systematic-review standards. No review protocol was registered: because the work was designed as a narrative review, it was not registered in PROSPERO (International Prospective Register of Systematic Reviews), which does not accept literature reviews, and it was not registered in any other registry. A structured literature search was conducted in MEDLINE (accessed through PubMed), Embase, and the Cochrane Central Register of Controlled Trials (CENTRAL), covering the period from 1 January 1990 to 30 September 2026. The search strategy combined controlled vocabulary (MeSH and Emtree terms) with free-text terms across three conceptual domains: micronutrients (micronutrients, trace elements, vitamin C, vitamin D, vitamin A, zinc, selenium, thiamine, copper, iron), critical illness (critical illness, sepsis, intensive care, multiple organ failure), and the gastrointestinal–microbial interface (gut failure, acute gastrointestinal injury, intestinal failure, intestinal permeability, gut barrier, dysbiosis, microbiota, bile acids, short-chain fatty acids, gut-associated lymphoid tissue, immune homeostasis). The three domains were combined with the Boolean operator AND, and terms within each domain with OR. No language restriction was applied at the search stage; non-English records were subsequently excluded if no English-language full text or translation was available. The full search strings for each database are reported in Appendix A. In addition, reference lists of recent narrative reviews and clinical practice guidelines on micronutrients and ICU nutrition, together with key articles on gut barrier function and microbiome-dependent nutrient metabolism, were manually screened to identify further eligible studies.

### 2.2. Study Selection and Flow

**Figure A1:**
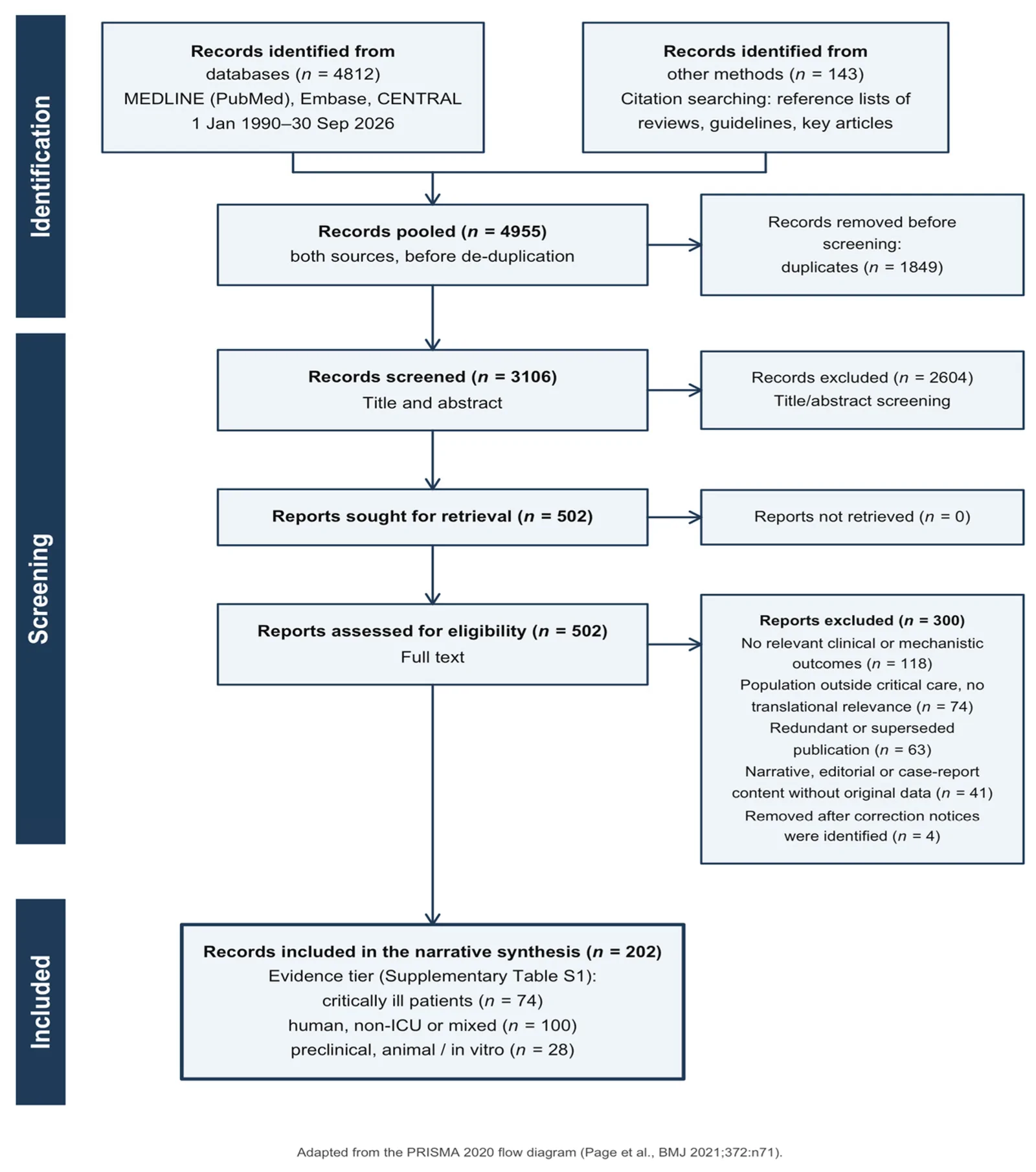
Flow diagram of study selection, adapted from the PRISMA 2020 template [36]. A total of 4812 records were identified through structured database searching of MEDLINE (PubMed), Embase, and CENTRAL (1 January 1990–30 September 2026), supplemented by 143 records identified through manual screening of reference lists of recent reviews, guidelines, and key mechanistic articles. After removal of 1849 duplicates (using Zotero 9.0.6), 3106 records were screened by title and abstract for relevance to micronutrient physiology or supplementation, gut integrity and microbial ecology, or immune function in critically ill or closely related populations; 2604 records were excluded at this stage. The remaining 502 articles were retained for full-text assessment. Of these, 300 were excluded based on predefined criteria, including absence of relevant clinical or mechanistic outcomes (*n* = 118), populations outside the critical care setting without translational applicability (*n* = 74), redundant data from overlapping cohorts or supersession by a more recent synthesis (*n* = 63), narrative, editorial, or case-report content without original data (*n* = 41), and records removed after correction notices were identified (*n* = 4). A total of 202 records were included in the final narrative synthesis (evidence tiers in Supplementary Table S1). When multiple publications addressed overlapping datasets or concepts, priority was given to the most comprehensive or most recent synthesis to avoid redundancy.

Study selection was performed in two stages by three authors (S.M., A.G., and F.V.): two reviewers (S.M. and A.G.) screened every record independently and in duplicate at both stages (title/abstract and full text), and disagreements were resolved by discussion with the third reviewer (F.V.), who acted as arbitrator. Records from all sources were imported into Zotero (version 9.0.6), which was used for record management and for removal of duplicates. In the first stage, titles and abstracts were screened for relevance to micronutrient physiology or supplementation, gut integrity and microbial ecology, or immune function in critically ill or closely related populations. Records clearly unrelated to critical illness or to the micronutrient–gut–immune axis, non-human studies without translational relevance, and reports lacking extractable outcome or mechanistic data were excluded. In the second stage, full texts of potentially relevant articles were assessed. Studies were selected based on relevance to critically ill populations or to the pathophysiological pathways under discussion, availability of clinically meaningful or mechanistically informative outcomes, and contribution to understanding of micronutrient assessment, route and timing of administration, or gut and microbiome status. Full-text exclusions were primarily due to absence of relevant clinical or mechanistic outcomes, populations outside the critical care setting without translational applicability, redundant data from overlapping cohorts, and narrative or editorial content without original data. When multiple publications addressed overlapping datasets or concepts, priority was given to the most comprehensive or most recent synthesis to avoid redundancy. In summary, records were eligible if they (i) concerned critically ill patients or the pathophysiological pathways linking micronutrients, gut function, microbiota, and immune function; (ii) reported clinically meaningful or mechanistically informative outcomes or provided the most comprehensive or most recent synthesis of a topic; and (iii) were available as an English-language full text. Guidelines, systematic reviews, and narrative reviews were nevertheless retained as secondary sources when they provided the most comprehensive or most recent synthesis of a pathway or clinical question. Overall, 4812 records were identified through database searching, with a further 143 records identified through manual screening of reference lists. After removal of duplicates, 3106 records were screened by title and abstract, of which 2604 were excluded. The remaining 502 articles were retained for full-text assessment; 296 were excluded after full-text evaluation based on predefined relevance and methodological criteria, and four further records were removed after correction notices (errata) were identified for them, leaving 202 records (primary studies and secondary sources) included in the final narrative synthesis. A flow diagram following the PRISMA 2020 template is provided (Figure A1, Appendix A). The results of the selection process are reported in Section 3. For each included record, study design, population or model (critically ill patients, other human populations, or animal/in vitro systems), and the manuscript sections it informs were tabulated (Supplementary Table S1). Six further references were added during revision to support methodological statements, epidemiological context, and topics raised by the reviewers; they are not counted among the 202 records.

### 2.3. Quality Appraisal and Synthesis Approach

Given the narrative nature of this review, a formal risk-of-bias assessment and meta-analytic pooling were not performed. An explicit evidence hierarchy was used to prioritize sources during selection and synthesis, in decreasing order: (1) randomized controlled trials; (2) large prospective or observational ICU cohorts; (3) mechanistic studies conducted in humans; and (4) animal and in vitro studies. Animal and in vitro data were retained when they clarified pathophysiological pathways not yet addressed in clinical settings and are identified as such throughout the text. Given the rapidly evolving literature, studies published in the last 10 years were preferentially cited when available, while older landmark mechanistic work was retained where no contemporary equivalent existed. Evidence was appraised qualitatively, with particular emphasis on study design, population characteristics, route and timing of micronutrient administration, the biomarker used to define micronutrient status, and the presence or absence of any assessment of gut integrity or microbiome composition. The structure and interpretation of the review were guided by SANRA principles, with an emphasis on transparency in methodology, balanced interpretation of the evidence, and integration of physiological and clinical perspectives.

To make the strength of evidence explicit, each included record was assigned to one of three evidence tiers according to the population or model on which its evidence rests: tier 1, critically ill patients (direct ICU evidence); tier 2, human studies outside the ICU or mixed human and mechanistic evidence (indirect evidence); and tier 3, animal or in vitro studies (preclinical evidence) (Supplementary Table S1). In the text and in Table 1, statements are accordingly qualified with four labels: “demonstrated in critically ill patients” (tier 1 evidence); “supported mainly by mechanistic or non-ICU human evidence” (tier 2); “supported mainly by animal or in vitro evidence” (tier 3); and “hypothesized in critical illness” (a plausible extrapolation that has not been tested in critically ill patients). Because no formal risk-of-bias tool was applied and no certainty-of-evidence grading was performed, these labels describe the type and setting of the evidence, not its methodological quality (see Section 4).

### 2.4. Use of Artificial Intelligence Tools

During the preparation of this manuscript, the authors used Claude Opus 5.5 (Anthropic) for language editing and for assistance in drafting the text in appropriate scientific English. Claude Opus 5.5 was also used to prepare the prompts for the figures generated with FigureLabs (AI-based scientific figure generator; web application, https://www.figurelabs.ai/, accessed in 2026) and, in the revised version, to assist with the R script (R version 4.5.1) that generates the PRISMA flow diagram (Figure A1) and with the draft classification of the records in Supplementary Table S1. Claude Sonnet 5.5 (Anthropic) assisted with the technical editing of the manuscript files in the revised version (renumbering of references and updating of counts) and with the assembly of the figure on clinical implications and research priorities (Section 3.7) from icons generated with FigureLabs. The AI tools were not used for the literature search, study selection, or appraisal of the evidence, nor to generate the scientific content, interpretation, or conclusions. All AI-assisted content was reviewed and edited by the authors, who take full responsibility for the content of this publication.

## 3. Main Body

Literature search results and evidence base: The database searches identified 4812 records, and the screening of reference lists identified a further 143 records (4955 in total). After removal of 1849 duplicates, 3106 records were screened by title and abstract and 2604 were excluded. Of the 502 full-text reports assessed, 300 were excluded (no relevant clinical or mechanistic outcomes, *n* = 118; populations outside the critical care setting without translational applicability, *n* = 74; redundant or superseded publications, *n* = 63; narrative, editorial, or case-report content without original data, *n* = 41; four records removed after correction notices were identified, *n* = 4), leaving 202 records in the synthesis (Figure A1, Appendix A). By evidence tier (Supplementary Table S1; percentages rounded), 74 records (37%) concerned critically ill patients, 100 (50%) human populations outside the ICU or mixed human and mechanistic evidence, and 28 (14%) animal or in vitro studies. By design, 104 records (51%) were narrative or expert reviews, 33 observational studies, 15 randomized controlled trials, 10 systematic reviews or meta-analyses, 6 guidelines or consensus statements, 31 experimental or laboratory studies, and 3 other publications (an editorial, a study protocol, and a pharmacovigilance analysis). Primary clinical studies in critically ill patients with reported results therefore numbered 37 (14 randomized trials and 23 observational studies). This composition reflects the aim of the review, which integrates physiology, microbiology, immunology, and clinical nutrition, and it has a consequence that is made explicit throughout: direct ICU evidence is concentrated in a limited number of trials and cohorts (Section 3.5), whereas most gut-specific mechanisms rest on non-ICU, animal, or in vitro evidence.

### 3.1. Micronutrient Deficiencies in Critical Illness: The Epidemiological Landscape

#### 3.1.1. Prevalence and Clinical Significance

Deficiencies of vitamins and trace elements are documented across virtually all ICU populations, independent of diagnosis [1,2]. A recent prospective study found significantly lower plasma selenium, beta-carotene, vitamin C, and vitamin E in critically ill patients versus healthy controls, already at admission [4]. In a COVID-19 ICU cohort, deficiency rates of vitamins A, B6, and D, plus zinc and selenium, reached 50–100%, with SOFA scores negatively correlating with vitamin D and selenium levels [37]. Thiamine deficiency affects up to 70% of septic shock patients and is mechanistically linked to lactate accumulation and mitochondrial dysfunction [38]. Magnesium depletion is nearly universal, worsened by polyuria, diuretics, CRRT, and gastrointestinal losses [39,40]. Critically, these low circulating levels are not static: plasma concentrations of selenium, zinc, vitamin C, and vitamin D fall progressively with each increment of C-reactive protein, with the steepest declines occurring above a CRP of 40 mg/L, meaning the degree of inflammation, not just baseline nutritional status, drives the apparent deficiency [32].

#### 3.1.2. The Translational Paradox

Although numerous observational studies associate micronutrient deficiencies with adverse clinical trajectories [3,32], interventional trials aimed at correcting these abnormalities as a simple problem of insufficient replenishment consistently fail to demonstrate meaningful improvements in patient outcomes [32]. This discrepancy challenges the assumption that restoring plasma concentration in critically ill patients necessarily restores biological function.

Intravenous supplementation of vitamin C, thiamine, vitamin B12, vitamin K, selenium, and iron can increase systemic availability, reliably normalizing circulating levels, yet these biochemical corrections do not necessarily translate into improved survival or organ support [32]. This dissociation between biochemical correction and clinical outcome may reflect differences in timing, dose, tissue distribution and utilization, disease phenotype, or target-organ function [41].

For the enteral-dominant group, the adequacy of intestinal absorption during gastrointestinal dysfunction remains insufficiently characterized. The trials mentioned above still rely on a gut absorption that may be structurally and functionally compromised, and the standard ICU continuous enteral delivery has not been systematically validated for micronutrient absorption against the bolus kinetics typically used to study transporter-mediated absorption [12]. For enterally delivered micronutrients, an important unresolved variable is not only the administered dose but the fraction that is effectively absorbed during gastrointestinal dysfunction [42,43].

A third, immunological layer compounds both groups. As summarized in Table 1, several micronutrients exert important biological effects at the intestinal epithelial and immune-cell level, where their activity depends on local uptake, transporter and receptor expression, tissue distribution, and intracellular metabolism [44,45]. Critical illness may disrupt these processes through mucosal inflammation, altered transporter/receptor expression, dysbiosis, edema, and impaired splanchnic perfusion [46,47]. Thus, restoration of circulating concentrations may not necessarily restore local biological activity. For most micronutrients, this immunological layer is supported mainly by mechanistic or non-ICU evidence and remains hypothesized in critical illness (Table 1).

Thus, gut failure should not be considered a universal explanation for negative supplementation trials. Rather, it may represent an unmeasured effect modifier, particularly for enterally delivered micronutrients and for interventions whose biological effects depend on intact mucosal or immune function. To the best of our knowledge, none of the trials identified by our search tested gut phenotype as an effect modifier of micronutrient supplementation in critically ill patients; this remains a hypothesis (Section 3.5).

**Table 1:** Gut-dependent determinants of micronutrient availability and biological function during critical illness. Multiple mechanisms of critical illness may affect micronutrient absorption, systemic availability, tissue delivery, cellular utilization, and immune function. The table summarizes micronutrient-specific gut physiology, potential alterations associated with critical illness and gut failure, relevant biological functions, implications for supplementation, and the current strength of direct ICU evidence. Evidence labels in the sixth column follow the definitions in Section 2.3.

Micronutrient/ Group	Gut-Dependent Physiology	Critical Illness/ Gut Failure	Immune/ Biological Relevance	Implications for Supplementation	Strength of Direct ICU Evidence	Representative References
Vitamin C	Intestinal SVCT-mediated uptake; saturable absorption	Inflammation, oxidative stress, increased cellular demand and altered distribution; enteral absorption may be limited	Antioxidant defense, endothelial integrity, leukocyte and immune function	IV administration bypasses absorption, but plasma correction does not ensure tissue repletion or clinical benefit	Demonstrated in critically ill patients (IV RCTs: no consistent benefit); gut-specific effect modification: hypothesized (untested).	[31,32,48,49,50]
Vitamin D	Dietary vitamin D2/D3 (skin synthesis is the other main source). Fat-soluble vitamin requiring bile-acid-dependent micellar solubilization and appropriate luminal pH. Apical uptake involves SR-BI, CD36, NPC1L1, and ABCA1	Gut failure may impair bile flow and enterohepatic bile-acid circulation. Bile-acid deficiency or deconjugation and altered luminal conditions destabilize micelles and reduce absorption	Barrier integrity, antimicrobial-peptide expression, innate and adaptive immunity	IV replacement bypasses intestinal uptake and may restore circulating concentrations, but it may not restore enterocyte processes or gut-localized vitamin activity	Demonstrated in critically ill patients (systemic repletion RCTs); gut-specific effects: mechanistic/non-ICU evidence, untested in the ICU.	[31,32,51,52,53,54,55,56,57,58,59,60]
Vitamin E	Dietary tocopherols; intestinal tocopherol uptake depends on both NPC1L1 and SR-BI. Fat-soluble vitamin requiring bile-acid-dependent micellar solubilization and appropriate luminal pH. Apical uptake involves SR-BI, CD36, NPC1L1, and ABCA1	Impaired bile flow, disrupted enterohepatic bile-acid circulation, and bile-acid deficiency or deconjugation impair micelle stability and promote steatorrhea and vitamin loss	Membrane antioxidant protection, regulation of lipid peroxidation, and immune function	Parenteral administration bypasses luminal and enterocyte handling. Tissue availability may differ from circulating concentrations, and restoration of gut-specific processes is uncertain	Limited direct ICU evidence (only within antioxidant combinations); gut mechanisms: mechanistic/non-ICU evidence.	[33,51,52,53,54,55,58,61]
Vitamin A/retinoids	Fat-soluble vitamin requiring bile-acid-dependent micellar solubilization at an appropriate luminal pH. Apical uptake of preformed retinol appears carrier-mediated, but no membrane transporter has been identified; broad-specificity lipid transporters (SR-BI, CD36, NPC1L1) mediate uptake of provitamin A carotenoids	Impaired bile flow, disrupted enterohepatic bile-acid circulation, altered luminal pH, and bile-acid deficiency or deconjugation destabilize micelles, causing steatorrhea and reduced uptake	Epithelial differentiation, barrier integrity, IgA responses, lymphocyte gut homing, and Treg/Th17 balance	IV replacement bypasses micellar solubilization, membrane uptake, and enterocyte processing. It may correct systemic deficiency without restoring enterocyte metabolic pathways supporting mucosal immunity	Mainly mechanistic/non-ICU evidence (largely pediatric intestinal failure); sparse direct ICU evidence.	[51,52,53,54,55,56,57,58,61,62,63,64,65]
Vitamin B1/thiamine	Transporter-mediated intestinal absorption; saturable uptake	Increased metabolic demand, inflammation, altered distribution and reduced intake	Mitochondrial energy metabolism, carbohydrate oxidation and immune-cell function	IV administration bypasses absorption but not intracellular utilization	Demonstrated in critically ill patients, largely in combination trials (thiamine effect not isolated); gut-specific effect: hypothesized.	[32,48,66,67,68,69]
Vitamin B12	Intrinsic-factor-dependent processing and terminal ileal absorption	Reduced intake, altered GI function and impaired ileal absorption may reduce availability	DNA synthesis, methylation, hematopoiesis and cellular metabolism	Enteral absorption may be unreliable in severe GI dysfunction; functional status may not be captured by serum B12 alone	Limited direct ICU evidence; available trials test pharmacological (H2S-scavenging) rather than nutritional dosing; gut-related effects: hypothesized.	[33,70]
Zinc	Absorption is mediated by ZIP-family importers, principally ZIP4, and ZnT-family exporters. Uptake depends on luminal availability, transporter expression, and epithelial integrity	Inflammatory cytokines modulate ZIP expression and can reduce luminal zinc uptake. Dysbiosis, epithelial injury, and altered zinc redistribution may further limit enteral delivery	Cofactor in more than 300 enzymatic reactions; epithelial barrier function, innate signaling, T-cell differentiation, and adaptive immunity	Low plasma zinc may indicate inflammation-driven redistribution as well as deficiency Enteral response may be variable when transporter expression or epithelial integrity is impaired	Mainly mechanistic and observational evidence (ICU associations; transporter data non-ICU/preclinical); no gut-stratified ICU supplementation trial.	[32,46,71,72,73,74]
Selenium	Intestinal absorption; incorporation into selenoproteins; plasma Se/selenoprotein P incompletely reflect tissue status	Inflammation alters hepatic selenoprotein synthesis and redistribution; gut-specific malabsorption less established	Glutathione peroxidase/thioredoxin systems; redox and immune regulation	Low plasma Se may reflect redistribution rather than isolated deficiency; gut-mediated treatment failure uncertain	Strong inflammation/redistribution evidence (observational ICU and animal data); gut-specific malabsorption: hypothesized.	[75,76,77,78]
Iron	Duodenal absorption via DMT1/ferroportin; regulated by hepcidin	Inflammation increase hepcidin and alters iron flux; reduced intake and dysbiosis may contribute	Hemoglobin synthesis, mitochondrial metabolism, oxygen transport and host–microbial interactions	Functional iron deficiency may coexist with adequate stores; ferritin/serum iron difficult to interpret during inflammation	Clinical/mechanistic evidence, mostly non-ICU (hepcidin biology); gut-specific evidence limited (hypothesized).	[65,79,80]
Copper	Intestinal uptake and systemic regulation of copper distribution	Inflammation, altered intestinal handling, nutritional deprivation and metal competition	Redox enzymes, mitochondrial function, connective tissue, hematopoiesis and immunity	Circulating copper may not reliably reflect tissue status; gut-specific ICU evidence limited	Limited direct ICU evidence; gut-specific effects: hypothesized.	[33,81,82]
Magnesium	Absorbed through passive paracellular and active transcellular pathways. Most total uptake occurs in the small intestine; TRPM6/TRPM7 mediate regulated transcellular absorption, particularly in the distal intestine and colon	Diarrhea, gastrointestinal losses, reduced intake, diuretics, renal replacement therapy, inflammation, and loss of distal absorptive capacity may contribute to deficiency	Enzymatic reactions, ATP metabolism, membrane stability, neuromuscular function, electrophysiology, and immune regulation	Gut failure is one of several causes of deficiency. Replacement should account for renal and extracorporeal losses; IV therapy bypasses absorption but does not restore regulated intestinal magnesium handling	Strong physiological evidence, mainly non-ICU; limited gut-specific ICU evidence (hypothesized).	[39,40,83,84,85]
Phosphate	Intestinal absorption includes passive paracellular uptake and sodium-dependent transcellular transport, particularly through NaPi-IIb	Enteral deprivation and inflammation suppress NaPi-IIb-mediated absorption. Reduced intestinal uptake may contribute to hypophosphatemia and refeeding syndrome in critically ill patients	ATP production, phosphorylation reactions, membrane phospholipids, nucleic acids, oxygen delivery through 2,3-DPG, and neuromuscular function	Serum phosphate reflects intake, absorption, renal handling, and intracellular redistribution. During refeeding, replacement must be integrated with cautious caloric advancement and monitoring of phosphate, potassium, and magnesium	Strong physiological and refeeding relevance; transporter-targeted strategies: hypothesized, with limited ICU evidence.	[86,87,88]
Vitamin K	Dietary phylloquinone (K1) plus menaquinones (K2) synthesized by colonic bacteria (Section 3.3.2). Fat-soluble vitamin requiring bile-acid-dependent micellar solubilization and appropriate luminal pH. Apical uptake involves broad-specificity lipid transporters, including SR-BI, CD36, NPC1L1, and ABCA1	Gut failure, impaired bile flow, disrupted enterohepatic bile-acid circulation, and bile-acid deficiency or deconjugation may reduce absorption. Antibiotic-associated disruption of microbial vitamin K production may contribute	Coagulation, bone metabolism, vascular biology, and cellular signaling	IV replacement bypasses micellar uptake and can correct coagulation-related deficiency, but it may not restore intestinal handling or gut-localized biological activity	Clinical evidence supports correction of deficiency; gut-specific effects: mechanistic/non-ICU, with limited ICU evidence.	[51,52,53,54,55,58]

Abbreviations: ABCA1, ATP-binding cassette transporter A1; ATP, adenosine triphosphate; CD36, cluster of differentiation 36; 2,3-DPG, 2,3-diphosphoglycerate; DMT1, divalent metal transporter 1; DNA, deoxyribonucleic acid; GI, gastrointestinal; H2S, hydrogen sulfide; ICU, intensive care unit; IgA, immunoglobulin A; IV, intravenous; NaPi-IIb, sodium-dependent phosphate cotransporter IIb; NPC1L1, Niemann-Pick C1-like 1; RCT, randomized controlled trial; Se, selenium; SR-BI, scavenger receptor class B type I; SVCT, sodium-dependent vitamin C transporter; Th17, T helper 17 cell; Treg, regulatory T cell; TRPM6/TRPM7, transient receptor potential melastatin 6 and 7; ZIP, Zrt/Irt-related protein; ZIP4, Zrt/Irt-related protein 4; ZnT, zinc transporter.

### 3.2. Gut Failure in Critical Illness: A Primer

The ESICM defines Acute Gastrointestinal Injury (AGI) as GI malfunction due to critical illness, graded from grade I (self-limiting increased risk of dysfunction) through grade II (dysfunction requiring intervention) and grade III (failure refractory to intervention) to grade IV (immediately life-threatening failure with impact on distant organ function and worsening MODS/shock). Across several cohorts, AGI affects roughly 40% of ICU patients, and grade III–IV AGI independently predicts mortality (HR 1.65 for 60-day mortality vs. grades I–II), with substantially greater mortality in grades III–IV than I–II [29,30,89].

This severity construct is inherently multidimensional rather than motility-based: Padar et al. operationalized gastrointestinal failure as ≥3 of six symptoms (high gastric residual volume, absent bowel sounds, vomiting, diarrhea, distension, GI bleeding), finding it in 10.4% of patients and an independent predictor of 90-day mortality [90]. Soranno et al. described dysbiosis, increased permeability with translocation, epithelial cell death, dysregulated gut immunity, and splanchnic vasoconstriction as jointly perturbed elements that drive and perpetuate multiorgan dysfunction [13]. Mortality seems similar between grades I–II and between III–IV, suggesting that a simpler dichotomy (dysfunction vs. failure) may outperform the four-tier system, and the framework has since evolved into the additive Gastrointestinal Dysfunction Score (GIDS), for which no biomarker, including citrulline or intestinal fatty acid-binding protein, has yet proven reliably useful for grading [89,91,92]. ESPEN complements this severity framework with a functional typology of Intestinal Failure (IF), defining acute, self-limiting type I, prolonged type II requiring weeks of parenteral support, and chronic type III requiring long-term IV supplementation. Together AGI/GIDS and IF frame gut failure as both a severity continuum and an absorptive threshold below which IV supplementation becomes mandatory [93].

Multiple insults commonly coexist in the ICU and may converge on the intestinal epithelium, microcirculation, motility, and microbial ecosystem. Shock-related hypoperfusion, systemic inflammation, vasopressor exposure, antibiotic-associated dysbiosis, enteral deprivation, and major surgery can therefore act synergistically to impair gut integrity and function (Table 2) [11,89,94,95,96,97,98].

Mechanistically, gut failure comprises at least four interacting components: epithelial barrier disruption, increased paracellular permeability, accelerated enterocyte apoptosis, and microbiome dysbiosis [99]. The epithelial barrier is maintained by a network of tight junction proteins—claudins, occludins, and zonula occludens—that regulate paracellular transport [100]. In critical illness, ischemia–reperfusion injury, systemic inflammation, and endogenous cytokines (particularly TNF-α and IL-6) disrupt tight junction assembly, leading to increased intestinal permeability and bacterial translocation [12,101]. Plasma markers of tight junction disruption—occludin, claudin-1, tricellulin, and zonulin—are elevated on admission in critically ill trauma patients and correlate with lactate, CRP, APACHE II, and SOFA scores [102]; plasma occludin and ZO-1 show analogous severity-dependent elevations in sepsis [103], and zonulin tracks nutrition risk in mixed ICU cohorts [104]. These markers remain investigational, lack gut specificity, and are limited by assay validation concerns, particularly for zonulin.

**Table 2:** Converging mechanisms contributing to gut failure during critical illness: Multiple insults commonly coexist in critically ill patients and may converge on the intestinal epithelium, microcirculation, motility, and microbial ecosystem. The table summarizes the principal mechanisms through which shock, systemic inflammation, vasopressor exposure, antibiotic therapy, enteral deprivation, and major surgery may impair intestinal integrity and function. These mechanisms may act simultaneously and interact, potentially contributing to impaired micronutrient absorption and altered intestinal immune function.

Insult/Mechanism	Principal Intestinal Effects	Potential Consequences for Gut Function	Representative References
Ischemia–reperfusion	Splanchnic vasoconstriction → reduced mucosal perfusion → enterocyte hypoxia, ROS generation, apoptosis	Epithelial injury, barrier disruption, impaired absorption; ↑ plasma I-FABP and L-FABP. Possible link to micronutrient handling: reduced absorptive surface and transporter capacity (Section 3.3.1).	[89,94]
Systemic inflammation/sepsis	Pro-inflammatory cytokines → caspase-3 activation, enterocyte apoptosis, ↓ occludin and claudin-1 expression	Loss of epithelial integrity, increased permeability, impaired barrier and immune function. Possible link to micronutrient handling: cytokine-driven changes in ZIP/ZnT expression and inflammatory redistribution of zinc, selenium, and iron (Section 3.3.1 and Section 3.3.3).	[102,103]
Antibiotic exposure	Rapid depletion of commensal bacteria and altered microbial ecology	Dysbiosis, altered microbial metabolism, impaired host–microbiome interactions and potentially nutrient handling. Possible link to micronutrient handling: loss of microbial vitamin K, folate, and SCFA production and of bile-acid transformation (Section 3.3.2 and Section 3.3.4).	[95,105,106,107]
Enteral deprivation	Loss of luminal trophic stimulation → villous atrophy and reduced mucosal blood flow	Reduced absorptive surface and epithelial function; delayed recovery of gut integrity. Possible link to micronutrient handling: reduced luminal stimulation of epithelial and GALT function (Section 3.3.1 and Section 3.4.5).	[11,96]
Vasopressor exposure	Norepinephrine/vasopressin-mediated splanchnic vasoconstriction → persistent or recurrent mucosal hypoperfusion	Ischemic epithelial injury and impaired barrier function, particularly in vulnerable patients. Possible link to micronutrient handling (hypothetical): reduced mucosal perfusion may limit uptake of enterally delivered micronutrients (Section 3.3.1).	[94,97]
Major surgery	Transient alterations in intestinal perfusion; ischemia–reperfusion, systemic inflammation, impaired motility, glycocalyx/barrier injury, delayed enteral exposure	Postoperative gut dysfunction, impaired barrier integrity and delayed restoration of enteral function. Possible link to micronutrient handling: delayed enteral exposure, relevant to the timing of enteral micronutrient delivery (Section 3.6.3).	[108,109]
Perioperative medications and inflammation	Antibiotics, opioids, and surgical inflammatory responses alter motility, microbiota, and epithelial function	Dysbiosis, impaired motility, barrier dysfunction and potentially prolonged gut failure. Possible link to micronutrient handling: antibiotic- and opioid-associated dysbiosis (Section 3.3.2).	[98]

Abbreviations: GALT, gut-associated lymphoid tissue; I-FABP, intestinal fatty acid-binding protein; L-FABP, liver fatty acid-binding protein; ROS, reactive oxygen species; SCFA, short-chain fatty acid; ZIP, Zrt/Irt-related protein; ZnT, zinc transporter. Arrows denote the direction of change or causal progression: → leads to/results in; ↑ increased; ↓ decreased.

As illustrated in Table 3, no single biomarker currently provides a validated stand-alone measure of gastrointestinal function in critically ill patients; clinical scores, bedside imaging, and selected blood or stool markers instead capture complementary domains of gut dysfunction.

### 3.3. How Gut Failure May Disrupt Micronutrient Homeostasis

Evidence note for Section 3.3 and Section 3.4: That critical illness alters the gut barrier, the epithelium, and the microbiome is documented in critically ill patients (Section 3.2). By contrast, most of the specific pathways that link these alterations to micronutrient absorption, metabolism, and immune function derive from mechanistic studies, from human populations outside the ICU (for example inflammatory bowel disease, intestinal failure, and short bowel syndrome), and from animal or in vitro models. Each subsection therefore states the level of evidence on which its main claims rest, using the four labels defined in Section 2.3: demonstrated in critically ill patients; supported mainly by mechanistic or non-ICU human evidence; supported mainly by animal or in vitro evidence; and hypothesized in critical illness.

#### 3.3.1. Absorption Failure: Transporter Dysfunction and Villous Atrophy

**Figure 1:**
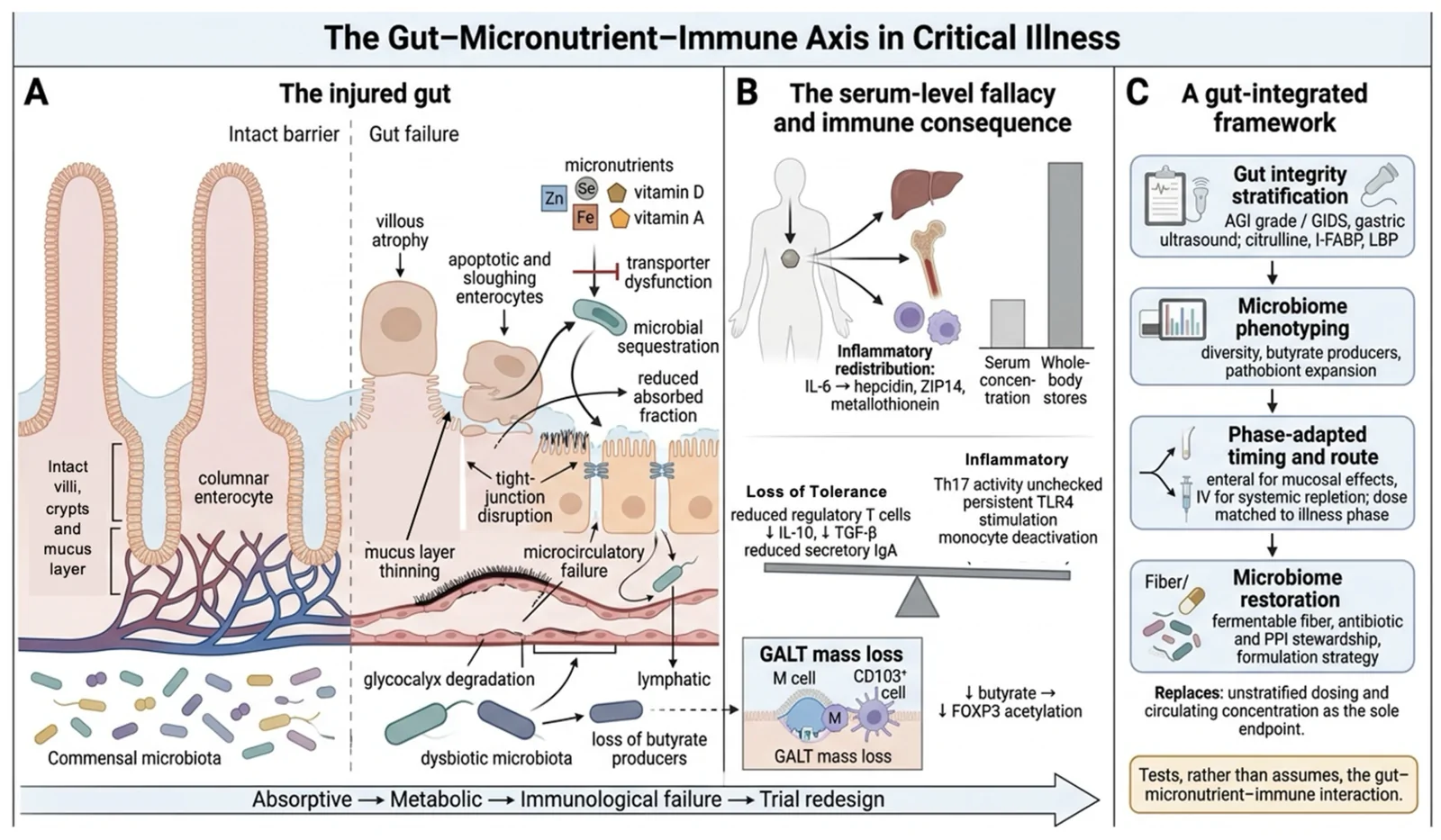
The gut–micronutrient–immune axis in critical illness. (**A**) Intact intestinal mucosa (**left**) compared with the injured gut in critical illness (**right**). Gut failure combines villous atrophy and enterocyte loss, tight-junction disruption, mucus layer thinning, endothelial glycocalyx degradation, microcirculatory failure, and dysbiosis with depletion of butyrate producers [11,12,13,89,94,118]. Enterally delivered micronutrients (zinc, selenium, iron, vitamin D, vitamin A) therefore meet three competing fates: blocked uptake through transporter dysfunction, diversion into the dysbiotic bacterial community through microbial sequestration, and a reduced absorbed fraction reaching the host [4,46,71,72,84,85]. (**B**) Systemic inflammation decouples circulating micronutrient concentrations from whole-body stores, as interleukin-6-driven hepcidin, ZIP14 and metallothionein responses redistribute trace elements and fat-soluble vitamins from plasma to liver, bone marrow and immune cells; low serum levels therefore need not indicate true depletion [8,75,76,79,119,120,121]. The paired bars are schematic and illustrative rather than measured. Below, loss of luminal butyrate reduces FOXP3 acetylation and regulatory T-cell induction, while gut-associated lymphoid tissue atrophies and secretory immunoglobulin A falls; bacterial translocation into mesenteric lymphatics sustains Toll-like receptor 4 stimulation, shifting the mucosal balance from tolerance toward unopposed T helper 17 activity and, subsequently, monocyte deactivation with reduced human leukocyte antigen-DR expression [15,122,123,124,125,126,127,128,129,130,131,132,133,134,135,136,137]. (**C**) Proposed gut-integrated framework, comprising gut integrity stratification, microbiome phenotyping, phase-adapted timing and route selection, and microbiome restoration, intended to replace unstratified dosing and reliance on circulating concentration as the sole endpoint [27,89,96,110,111,113,138,139,140,141,142,143,144,145,146,147,148,149,150,151,152]. Arrows indicate the causal or sequential relationships (→ leads to/induces) and the direction of change (↓ decreased). Abbreviations: AGI, acute gastrointestinal injury; CD103, cluster of differentiation 103; FOXP3, forkhead box P3; GALT, gut-associated lymphoid tissue; GIDS, Gastrointestinal Dysfunction Score; I-FABP, intestinal fatty acid-binding protein; IgA, immunoglobulin A; IL, interleukin; IV, intravenous; LBP, lipopolysaccharide-binding protein; PPI, proton pump inhibitor; TGF-β, transforming growth factor beta; Th17, T helper 17; TLR4, Toll-like receptor 4; ZIP14, Zrt/Irt-related protein 14.

Gut failure may impair the enteral uptake pathway at multiple levels, potentially creating a mismatch between the amount administered and the fraction effectively absorbed. In critical illness, villous atrophy and enterocyte loss reduce the functional absorptive surface and impair both carrier-mediated and passive nutrient uptake [11,12,13,118]. This effect may be particularly relevant for micronutrients whose absorption depends on tightly regulated intestinal transport, such as magnesium and zinc. Inflammation can further modify transporter expression and intracellular handling, potentially reducing uptake independently of the administered dose [46,71,72,84,85] (Figure 1A).

Fat-soluble micronutrients may be additionally vulnerable to alterations in bile-acid availability, luminal lipid handling, and micellar solubilization [58,61]. Vitamins A, D, E, and K therefore depend on an intact intestinal environment that supports both luminal processing and epithelial uptake [51,52]. Critical-illness-associated changes in bile flow and enterohepatic bile-acid homeostasis may further compromise this process [53,54,55].

Enteral supplementation may therefore provide less effective exposure. Conversely, IV micronutrient replenishment bypasses these absorptive steps and may correct circulating concentrations, but it cannot be assumed to restore downstream determinants of biological activity, tissue delivery, cellular uptake, intracellular metabolism, or enterocyte-dependent immune function [51,56,57]. *Evidence level:* failure of enteral delivery to normalize plasma levels, with no relationship between intake and circulating concentrations, has been shown in critically ill patients (Section 1); transporter-level and bile-acid mechanisms are supported mainly by mechanistic, non-ICU, or preclinical evidence; and the quantitative contribution of malabsorption to supplementation failure remains hypothesized in critical illness.

#### 3.3.2. Microbiome-Dependent Metabolism

An often-neglected aspect of micronutrient homeostasis is the role of intestinal commensals. The human gut microbiome synthesizes or activates several essential nutrients, being a crucial part in their intestinal absorption (Figure 1A). Direct ICU evidence on these microbiome-dependent pathways is scarce: most of the data below derive from healthy volunteers, non-ICU patients, and animal models, and their relevance to micronutrient supplementation in critical illness is hypothesized rather than demonstrated. We therefore limit the discussion to nutrients and metabolites with a plausible link to micronutrient availability or immune function:

- Vitamin K2 (menaquinone): gut commensals (Bacteroides, Prevotella, Eubacterium lentum, Bacteroides fragilis, Lactococcus lactis, and Propionibacterium species) synthesize menaquinones in the large intestine, and fecal menaquinone content tracks with microbiota composition [153,154,155]. The administration of Broad-spectrum antibiotics lowers hepatic vitamin K levels and impairs vitamin k dependent osteocalcin carboxylation, confirming the importance of bacterial synthesis [122].
- Vitamin B12: dietary B12 is absorbed in the terminal ileum via intrinsic factor, and, although a subset of bacteria and archaea synthesize cobalamin de novo, most gut microbes are net consumers; in fact, over half encode cobamide transporters and can strip B12 from intrinsic factor [122,153], furthermore, these bacteria are located in the large intestine, where humans lack intrinsic factor-B12 luminal transporters. Therefore, dysbiosis alters luminal B12 dynamics primarily through microbial competition and cross-feeding [153].
- Folate: folate biosynthesis genes are ubiquitous across the microbiota’s genomes, with lactic acid bacteria (i.e., Lactobacillus and Bifidobacterium) producing folate for host’s enterocytes to assimilate [153,156]. The loss of these microbes might reduce luminal folate bioavailability, concurring to dietary deficiency during critical illness [156].
- Short-chain fatty acids (SCFAs): SCFAs, produced by fiber fermentation predominantly by Firmicutes (F. prausnitzii, Roseburia spp., Eubacterium rectale), yield butyrate that serves as the primary colonocyte energy substrate via the TCA cycle and β-oxidation, promotes tight-junction assembly through AMPK activation, and drives regulatory T-cell induction via HDAC inhibition and Foxp3 acetylation [122]. Loss of these SCFA producers, a defining feature of ICU dysbiosis, impairs all three functions simultaneously [157,158].
- Trimethylamine *N*-oxide (TMAO): TMAO is a gut microbiota-dependent metabolite formed when gut microbiota converts dietary choline, phosphatidylcholine, and L-carnitine to trimethylamine, which the liver oxidizes via flavin-containing monooxygenase 3 (FMO3) [159,160,161]. In non-ICU cohorts, elevated circulating TMAO has been associated with major adverse cardiovascular events [160,162,163,164,165]. Because no direct ICU evidence links TMAO to the response to micronutrient supplementation, this metabolite is mentioned only as an example of microbiota-dependent nutrient metabolism, and its cardiovascular implications are not discussed further.

#### 3.3.3. Inflammatory Redistribution: The Serum-Level Fallacy

Systemic inflammation profoundly decouples serum micronutrient concentrations from tissues, as most plasma vitamins’ and trace elements’ levels fall progressively even with modest CRP elevations [8,119]. As part of the acute-phase inflammatory response, metals and fat-soluble vitamins are actively redistributed from the circulation to the liver and immune/inflammatory cells, as a way to hinder bacterial growth [4,23,32].

For example, Zinc serum levels drop because IL-6 induces the hepatocyte zinc importer ZIP14, coupled with metallothionein for intracellular storage, shifting zinc into liver, bone marrow, and thymus without necessarily reducing whole-body stores [120]. Selenium behaves similarly since acute inflammation downregulates the hepatic selenoprotein-biosynthesis machinery and reprioritizes selenium toward retained intracellular selenoproteins at the expense of exported selenoprotein P, resulting in low plasma selenium that does not indicate true deficiency [75,76]. IL-6-induced hepcidin binds ferroportin and triggers its internalization and degradation, blocking iron excretion from enterocytes and macrophages, causing hypoferremia with elevated ferritin and hepcidin, often mistaken for absolute iron deficiency in the ICU [79,121] (Figure 1B).

This inflammatory redistribution implies that serum levels can normalize despite inadequate micronutrient availability at critical cellular targets, and conversely, that prescribing micronutrients aiming to correct “low” serum concentrations may oversaturate certain cellular compartments. *Evidence level:* Inflammation-driven falls in plasma zinc, selenium, iron, vitamin C, and vitamin D concentrations are demonstrated in critically ill patients and in other human populations (Section 3.1.1); the underlying signaling pathways (interleukin-6/ZIP14, hepcidin, selenoprotein regulation) derive mainly from animal and mechanistic studies, and the implication that normalized serum levels may coexist with inadequate cellular availability is hypothesized in critical illness.

#### 3.3.4. Enterohepatic Cycling Disruption and Bile Acid Dysmetabolism

Bile acids form micelles that solubilize dietary fat and the fat-soluble vitamins A, D, E, and K, and ~95% are actively reabsorbed in the terminal ileum via the apical sodium-dependent bile acid transporter (ASBT/SLC10A2) and return to the liver [53,166]. Efficient recycling therefore depends on intact biliary secretion, functional ileal ASBT, and microbial bile acid metabolism, since only ~5% of the pool is normally lost in feces [53,167]. After bile salt hydrolase-mediated deconjugation, colonic bacteria, mainly 7α-dehydroxylating Clostridia with contributions from Bacteroides, Eubacterium, and Ruminococcaceae/Lachnospiraceae, convert cholic and chenodeoxycholic acid (CDCA) into the secondary bile acids deoxycholic (DCA) and lithocholic acid (LCA) [122,168,169]. These secondary bile acids are key ligands for the farnesoid X receptor (FXR) and the G protein-coupled bile acid receptor TGR5 (GPBAR1). Downstream signaling works through FXR and TGR5. They are expressed on enterocytes and immune cells, regulate intestinal barrier integrity, motility, and immune tone. FXR suppresses NF-κB-driven cytokine production and improves barrier function, while TGR5 restrains NLRP3 inflammasome activation and promotes IL-10 and Treg-associated (Foxp3, TGF-β) responses [122,170].

Critical illness, through broad-spectrum antibiotics, altered nutritional intake, and hypoperfusion, depletes the commensal anaerobes, including the Clostridia/Lachnospiraceae that perform 7α-dehydroxylation. As a result, the primary-to-secondary bile acid ratio raises and impairs bile acid signaling [34,47,171]. As mentioned above, bile acids are required to solubilize and absorb fat-soluble vitamins. This dysmetabolism can reduce these micronutrients’ bioavailability regardless of enteral supplementation [53,172]. *Evidence level:* Bile-acid metabolism and its microbial regulation are supported mainly by mechanistic and non-ICU evidence (including a study in critically ill children and intestinal-failure cohorts); the effect on fat-soluble vitamin bioavailability in adult critical illness is hypothesized.

### 3.4. Immune Consequences of Combined Gut Failure and Micronutrient Dysfunction

#### 3.4.1. Zinc and Selenium as Innate Immune Regulators

Zinc is a structural or catalytic cofactor for hundreds of enzymes and numerous zinc-finger transcription factors for innate immunity [173]. Its best-characterized immunologic action is restraining NF-κB: zinc induces the zinc-finger protein A20 (TNFAIP3), which deubiquitinates upstream signaling intermediates to inhibit IKK/NF-κB and thereby suppress TNF-α and IL-1β production [73,174]. In zinc deficiency this brake is lost, NF-κB activation proceeds unchecked, and pro-inflammatory cytokine output rises. This is consistent with the inverse relationship between low plasma zinc and organ dysfunction/mortality in sepsis [74].

Selenium acts through a complementary redox mechanism. The selenoenzyme glutathione peroxidase clears hydrogen peroxide and lipid hydroperoxides, attenuating oxidative NF-κB activation; selenium-deficient macrophages show prolonged NF-κB nuclear translocation and increased iNOS/NO output, whereas selenium repletion suppresses this response and can favor M2/anti-inflammatory macrophage polarization [175,176]. In critical illness, low selenium correlates with greater oxidative damage, higher organ-failure scores, and mortality [74,77].

Both trace elements depend on transporter-mediated intestinal uptake that is impaired by mucosal injury, reduced enterocyte surface area, and dysregulated ZIP/ZnT expression in gut failure [46]. Their plasma levels are further lowered by acute-phase redistribution and are resistant to parenteral supplementation, as mentioned before [173]. *Evidence level:* The immunoregulatory actions of zinc and selenium are supported mainly by mechanistic evidence (cell-based and animal studies); in critically ill patients, low plasma concentrations have been shown to be associated with oxidative damage, organ dysfunction, and mortality (observational data), whereas causality and gut dependence are hypothesized.

#### 3.4.2. Vitamin D: Gut Absorption, VDR Signaling, and Microbiome Crosstalk

The immunologic reach of vitamin D extends well beyond calcium homeostasis: the vitamin D receptor (VDR) is expressed across innate and adaptive immune cells and in intestinal epithelium, where 1,25(OH)_2_D-VDR signaling regulates antimicrobial defense, barrier maintenance, and the Th1/Th17-to-Treg balance [177,178]. A central innate-immune output is direct, VDR-mediated transcription of the antimicrobial peptides cathelicidin (LL-37/CAMP) and β-defensin (DEFB4/hBD-2) through vitamin D response elements in their promoters, sustaining the mucosal antimicrobial barrier [179,180]. This axis is bidirectional since vitamin D deficiency is associated with fewer Bifidobacterium, Lactobacillus, and Akkermansia and a reduced Bacteroidetes-to-Firmicutes ratio while supplementation shifts the microbiota toward beneficial flora [157,181]. Conversely, VDR signaling maintains barrier integrity by transcriptionally regulating tight-junction proteins (e.g., CLDN2 is a direct VDR target) and by controlling epithelial apoptosis and autophagy [182,183]. Inflammation causes the loop to become self-reinforcing: epithelial VDR is downregulated and, although commensal-dependent, NF-κB-driven induction of colonic CYP27B1 (1α-hydroxylase) partially compensates by raising local 1,25(OH)_2_D. Loss of commensals impairs this microbiota-dependent activation and barrier signaling [184,185]. Clinically, this predicts that supplementing vitamin D in a patient with gut failure and dysbiosis may normalize circulating 25(OH)D without restoring VDR-mediated antimicrobial and barrier function at the mucosal level. This remains a hypothesis: the VIOLET trial is compatible with it, because a single high-dose enteral load rapidly corrected deficiency yet produced no mortality or clinical benefit in critically ill patients, but VIOLET did not assess gut function and cannot distinguish this explanation from alternatives such as timing, dose, or a lack of efficacy of vitamin D repletion irrespective of gut status [59,186]. *Evidence level:* The barrier and antimicrobial effects of vitamin D receptor signaling are supported mainly by in vitro, animal, and non-ICU human evidence; the ICU data are limited to systemic repletion trials (Section 3.5).

#### 3.4.3. Vitamin A, Retinoic Acid, and the Conditioning of Mucosal Tolerance

Vitamin A is distinctive among micronutrients because its active metabolite, all-trans retinoic acid (RA), is key in programming the GALT, Treg, IgA and barrier functions that are lost in intestinal failure. Dietary retinyl esters and carotenoids are absorbed proximally, converted to retinol, and oxidized by retinaldehyde dehydrogenases (RALDH) to RA, which signals through nuclear RAR/RXR receptors [187,188]. Because this depends on fat-soluble vitamins, it is affected by all the mechanisms mentioned above and is documented in intestinal failure studies [65,189].

Combined gut failure and VAD converge on four interlocking mechanisms: (1) RA from CD103^+^ dendritic cells acts with TGF-β to drive Foxp3^+^ Treg differentiation while suppressing Th17; in VAD this brake is released and Th1/Th17 activity goes unopposed [62,190]; (2) RA imprints the gut-homing receptors α4β7 and CCR9, required for effector and regulatory lymphocytes’ honing and diapedesis to the already GALT [188,191]; (3) RA directs B-cell class-switching to IgA, and VAD reduces secretory IgA, weakening frontline defenses against bacterial adherence and translocation [191,192]; and (4) RA maintains the barrier by regulating tight junctions, mucins (MUC2/MUC3), and antimicrobial defensins and by supporting IL-22-producing ILCs. Moreover, VAD increases permeability and shifts the microbiota’s composition toward dysbiosis (i.e., E. coli expansion, Lactobacillus loss) [63,193].

This suggests a self-reinforcing malabsorption–deficiency–infection cycle by analogy with zinc and selenium. The barrier defect is synergistic with zinc: simultaneous repletion of vitamin A and zinc normalized permeability and growth when neither solved the issue alone [64,65]. Furthermore, RA’s tolerogenic outputs are microbiota-dependent (dendritic-cell RALDH is TLR-induced), so correcting circulating retinol may not restore mucosal RA-driven Treg and IgA pathways in dysbiotic gut failure [62]. As with vitamin D and other trace elements, adequate systemic vitamin A does not guarantee a competent mucosal effector system. *Evidence level:* Supported mainly by non-ICU evidence (pediatric intestinal-failure cohorts) and by animal and mechanistic studies; hypothesized in adult critical illness.

#### 3.4.4. Iron and Copper: Redox-Active Trace Elements at the Host–Microbe Interface

Iron and copper are the trace elements most frequently deficient in intestinal failure after zinc and the fat-soluble vitamins. Iron deficiency is the single most common micronutrient deficit in pediatric cohorts, and copper deficiency affects up to roughly one-third of patients, reflecting impaired proximal absorption, mucosal surface loss, and ongoing gastrointestinal losses [65,189].

Iron is bidirectional in innate immunity: it shapes macrophage antimicrobial capacity and modulates NF-κB, HIF-1, STAT1, and Nrf2, while catalyzing Fenton-reaction reactive oxygen intermediates that amplify NF-κB-driven inflammation [80]. Iron deficiency impairs immune-cell function and warrants correction, yet excessive iron levels promote oxidative injury and favors Enterobacteriaceae expansion and translocation. For this reason, hepcidin-guided intravenous repletion is preferred in critical illness to confirm true deficiency and avoid indiscriminate loading [4].

Copper is an essential cofactor for Cu/Zn superoxide dismutase, cytochrome c oxidase, and ceruloplasmin, supporting antioxidant defense, respiration, iron mobilization, and immune-cell function [194]. Its deficiency, often precipitated or masked by high-dose zinc through competitive enterocyte uptake, causes neutropenia and impaired phagocytosis, directly compromising innate defense. Because copper and zinc interact during absorption, repletion of one must account for the other, reinforcing that micronutrient correction in gut failure requires coordinated, monitored dosing rather than isolated single-nutrient strategies [65,80]. *Evidence level:* Iron-deficiency and hepcidin biology and copper–zinc interactions are supported mainly by non-ICU and mechanistic evidence; their gut dependence in critical illness is hypothesized.

#### 3.4.5. SCFA Depletion, Regulatory T-Cell Loss, and GALT Dysfunction

Short-chain fatty acids are both colonocyte fuel and immune mediators with local and systemic reach, acting through GPCR signaling (GPR43/41, GPR109A) and histone deacetylase (HDAC) inhibition [123,124]. Butyrate is the best-characterized: by inhibiting HDACs it enhances acetylation of the FOXP3 locus, driving the differentiation and stability of colonic regulatory T-cells (Tregs) and IL-10 production, a mechanism central to mucosal tolerance and restraint of inflammatory activation [122,125] (Figure 1B). In gut dysbiosis, depletion of butyrate producers, such as Faecalibacterium prausnitzii, Roseburia intestinalis, and Eubacterium hallii, lowers this immunomodulatory pool, impairing Treg induction and destabilizing the tolerogenic mucosal milieu [123,126].

GALT, Peyer’s patches, mesenteric lymph nodes, isolated lymphoid follicles, and dispersed intraepithelial and lamina propria lymphocytes comprise the largest immune compartment in the body, holding roughly 75% of the body’s lymphocytes [127,128]. Its tolerogenic function depends on luminal antigen sampling via M cells and CD103^+^ dendritic cells, epithelial integrity, and SCFA-mediated conditioning of these cells toward Treg induction and IL-10/TGF-β output [129,130]. Loss of enteral nutrient delivery and SCFAs reduces GALT mass, lymphocyte numbers in Peyer’s patches, and secretory IgA, shifting the mucosal milieu from tolerogenic toward pro-inflammatory, thus supplemented micronutrients may encounter a compromised immune effector system regardless of circulating concentration [131]. *Evidence level:* Supported mainly by animal and in vitro studies, with human data from non-ICU populations; the contribution of SCFA depletion to micronutrient response in critical illness is hypothesized.

#### 3.4.6. Bacterial Translocation and Systemic Immune Exhaustion

The convergent endpoint of gut barrier failure, dysbiosis, and GALT dysfunction is microbial translocation, where gut-derived organisms serve as a major reservoir for secondary infection [132,133]. In septic shock, translocation peaks early on, worsening with AGI grade and progressive loss of microbiota diversity (i.e., expansion of Enterococcus and Enterobacteriaceae and depletion of butyrate producers) [15,134]. This chronic microbial stimulation sustains Toll-like receptor (particularly TLR4) activation, which, after an initial hyperinflammatory phase, drives a compensatory hyporesponsive state characterized by reduced monocyte HLA-DR, blunted LPS-induced cytokine output, and loss of monocyte metabolic plasticity and oxidative burst [135,136,137] (Figure 1B). In the ICU, this coupling of intestinal Enterobacteriaceae enrichment with hypofunctional, immature myeloid cells is directly associated with impaired host defense and nosocomial infection [15].

The therapeutic implication is that adequate circulating micronutrient concentrations do not guarantee a competent molecular target. Zinc’s restraint on NF-κB requires an NF-κB-competent monocyte/macrophage pool capable of transducing TLR4 signaling, the very signaling axis blunted in endotoxin tolerance [195,196]. Likewise, vitamin D-driven cathelicidin induction and barrier maintenance need a functional VDR signaling pathway, yet LPS downregulates epithelial VDR and chronic inflammation renders supplementation ineffective in restoring mucosal effector function [184,197]. Thus, translocation-driven immunoparalysis may render micronutrient targets functionally refractory even when the cofactor is present [15,137]. *Evidence level:* Associations among dysbiosis, translocation, immune hyporesponsiveness, and nosocomial infection have been demonstrated in critically ill patients; the proposal that these changes blunt the response to micronutrients is hypothesized and rests on in vitro and non-ICU evidence.

### 3.5. Why Might Trials Fail? The Micronutrient–Gut Interface Is Unmeasured

As summarized in Table 4, several well-powered RCTs testing micronutrient supplementation in the ICU have failed to demonstrate clinical benefit, with some reporting potential harm [32]. However, these trials were not designed to test whether gut integrity and microbial ecology modify micronutrient efficacy. Importantly, the trials in Table 4 differ in route (intravenous, enteral, or both), intervention, and population, and none assessed gut function as an effect modifier; the observations below are therefore hypothesis-generating.

REDOXS exemplifies this gap: 1223 mechanically ventilated patients with multiorgan failure received a combined antioxidant regimen (selenium, zinc, beta-carotene, vitamins E and C) by both enteral and intravenous routes, without stratification by epithelial integrity, absorptive capacity, or microbial state, and antioxidants conferred no benefit on 28-day mortality or any secondary endpoint [198].

Several important variables were largely unmeasured. First, patients with different degrees of gastrointestinal dysfunction were generally analyzed together, without systematic assessment of epithelial integrity or absorptive capacity. Second, several of the trials in Table 4 used the IV route (vitamin C in CITRIS-ALI, VITAMINS, and LOVIT; thiamine in VITAMINS; and part of the REDOXS regimen), which can correct circulating deficiency but bypasses the enterocyte–microbiome–immune interface and therefore cannot reproduce potential local effects of enteral micronutrients delivery including effects on mucosal integrity [12,64], SCFA-dependent Treg induction [123,124], and mucosal GALT conditioning [199,200]. For micronutrients whose primary targets are extraintestinal (e.g., systemic antioxidant enzymes), gut failure may still contribute indirectly by sustaining endotoxemia, microcirculatory shunting, and immunoparalysis but is unlikely to be the sole explanation for lack of benefit or harm [4]. Conversely, VITdAL-ICU and VIOLET used enteral vitamin D, but neither assessed gastrointestinal absorptive capacity. For the intravenous trials, impaired enteral absorption cannot explain the lack of benefit; any contribution of gut failure would have to operate through downstream mechanisms (for example, immune-cell responsiveness, endotoxemia, or microcirculatory dysfunction) and remains hypothetical.

Third, microbiome composition, dysbiosis severity, and antibiotic exposure were not incorporated into treatment stratification, despite their potential to alter nutrient metabolism, micronutrient availability, and host response.

Importantly, serum concentrations do not necessarily reflect tissue or intestinal bioavailability during critical illness because of inflammatory redistribution, altered binding proteins, and acute-phase reactant confounding. Moreover, enterally delivered micronutrients may be subject to microbial competition, particularly for iron and zinc, while tube-feeding logistics and nutrient–drug interactions may further reduce the effective dose reaching the absorptive surface [81,201]. Finally, supplementation was rarely aligned with the dynamic trajectory of immune dysfunction and gut recovery.

Thus, the landmark RCTs establish that broad micronutrient supplementation does not consistently improve outcomes at the population level, but they do not adequately address whether intestinal integrity, microbiome state, route of delivery, timing, and effective nutrient exposure determine individual response. This unmeasured gut–micronutrient interface therefore represents an important gap in the interpretation of existing trials and in the design of future studies [4,32]. Gut failure should therefore be regarded as a plausible but currently insufficiently tested modifier of the response to micronutrient supplementation, not as an established explanation for the negative results of previous trials.

### 3.6. Toward a Gut-Integrated Approach to Micronutrient Therapy

Testing whether gut integrity modifies the response to micronutrient therapy would require treating it not as a covariate to adjust for, but as a primary and dynamic variable in trial design. We propose, as a hypothesis-generating framework, four interlocking components: gut integrity stratification, microbiome phenotyping, phase-adapted timing and route selection, and microbiome restoration (Figure 1C).

#### 3.6.1. Serial Gut Integrity Monitoring as a “Gut Vital Sign”

Several blood biomarkers offer complementary windows on gut integrity: plasma citrulline reflects functional enterocyte mass and I-FABP reflects acute enterocyte injury, while lipopolysaccharide-binding protein (LBP) captures endotoxin burden and translocation [27,96,113]. However, prospective ICU data do not support their use: serial citrulline and I-FABP correlated poorly with clinical gastrointestinal dysfunction and are better regarded as admission prognostic markers than functionality markers [27,110,111]. Zonulin, though frequently proposed as a permeability readout, should be avoided because widely used assays cross-react with proteins of unclear barrier relevance; LBP (optionally with soluble CD14 or EndoCAb) is the better-validated translocation surrogate [113,202]. Fecal calprotectin is an attractive noninvasive candidate, being stable in stool and rising with ischemic mucosal injury and hypoxemia severity, but its ICU validation outside COVID-19 remains preliminary and is confounded by ICU diarrhea [115,116,203,204].

A more feasible approach employs a validated daily clinical score rather than a novel research-grade panel: the Gastrointestinal Dysfunction Score (GIDS) or AGI grading is scored from routine bedside data, is additive to SOFA for mortality prediction, and replaces the binary tolerant/intolerant labeling based on gastric residual volume alone [89,110,138]. This can be paired with bedside gastric ultrasound, which predicts gastrointestinal dysfunction better than serum biomarkers in the same cohort and is repeatable and radiation-free [111].

Within a research framework, citrulline, I-FABP, LBP, and fecal calprotectin may be used as complementary mechanistic or stratification variables, while clinical scores and bedside assessment remain the more feasible basis for serial gastrointestinal phenotyping [27,96,116,203].

#### 3.6.2. Microbiome Restoration as a Potential Adjunct to Micronutrient Therapy

Microbiome composition may influence micronutrient handling and immune response, but whether microbiome restoration improves the efficacy of micronutrient therapy in critically ill patients remains unproven.

Multi-strain Lactobacillus/Bifidobacterium probiotics showed early signals for reducing ventilator-associated pneumonia and length of stay, but the effect vanished in low-risk-of-bias trials [139,140], the large PROSPECT RCT found no VAP reduction [141], and the 2026 Surviving Sepsis Campaign now conditionally recommends against them in sepsis [142].

Selective digestive decontamination reduces VAP and improves survival in low-resistance settings Wittek [143], though the SuDDICU trial showed no mortality benefit and any effect on micronutrient handling via reduced LPS-driven hepatic redistribution remains an untested hypothesis [4,144].

Fecal microbiota transplantation is feasible and safe in early ICU data, restoring diversity and reducing multidrug-resistant-organism carriage [145], with dedicated ICU trials ongoing [146], yet whether it improves subsequent micronutrient bioavailability or reduces competitive iron/zinc sequestration has never been directly tested and represents a concrete trial question [147].

Prebiotic fibers can feed butyrate producers and support Treg-inducing conditions through FOXP3 acetylation [148], but a pilot trial in critically ill trauma patients showed context-dependent, sometimes detrimental effects—accelerated Bifidobacterium loss and Enterobacteriaceae expansion—driven by concurrent antibiotic administration, showing how a dysbiotic gut may divert substrate to pathobionts [149].

Beyond these, fermentable dietary fiber drives barrier support and M2 macrophage polarization with a low absolute risk of mesenteric ischemia and is now favored once enteral nutrition is tolerated [150], while synbiotics remain a mechanistically distinct but similarly uncertain option [140].

Antibiotic and proton-pump-inhibitor stewardship is arguably the highest-yielding intervention, since antibiotic exposure is the dominant driver that converts prebiotic responses from beneficial to harmful and induces butyrate-producer collapse [149,151]. Direct butyrate supplementation is a logical alternative when key producers are absent, though only preclinical data is available [148,151].

Finally, formulation strategy is itself part of restoration, since unabsorbed luminal iron feeds iron-scavenging pathobionts such that hepcidin-guided intravenous iron may bypass this provisioning [4,152], and because the microbiome broadly modulates the bioavailability of vitamins A, B, C, D, E, and K and of iron, zinc, calcium, and magnesium, microbiome state may be best framed as a candidate determinant of micronutrient response rather than a parallel target [147,205,206].

#### 3.6.3. Route Selection Beyond the IV/Enteral Binary: Toward Gut-Integrated Micronutrient Dosing

Micronutrient repletion may fail through two distinct, hypothesized mechanisms depending on the route chosen, and neither is captured by current guidelines.

1. Enteral failure would occur when the gut itself cannot absorb what is delivered. Gut failure may produce villous atrophy, downregulation of nutrient transporters, disrupted bile acid metabolism, and dysbiosis—a cascade that may impair both absorption and local micronutrient processing. Reaching target enteral nutrition (EN) volumes says nothing about whether transporter-mediated absorption has normalized; caloric tolerance and micronutrient uptake are not the same physiological event.
2. Parenteral failure would occur further downstream, at the level of utilization rather than delivery. Vitamins and trace elements act clinically largely by modulating immune cells and inflammatory signaling—NF-κB activity, redox balance, vitamin D receptor (VDR) signaling, and the short-chain fatty acid (SCFA)–regulatory T cell (Treg) axis. Because gut-associated lymphoid tissue (GALT) represents a dominant fraction of total immune mass, gut failure may compromise this signaling architecture directly: GALT becomes exhausted, chronic low-grade endotoxemia from dysbiosis persists, and the mucosal barrier breaks down. The resulting immune cells may be either hyperactivated and then exhausted (immunoparalysis) or functionally misprogrammed. In this state, intravenous (IV) micronutrients can reach normal plasma concentrations yet may fail to restore immune function, because their target—mucosal and systemic immune cells—is itself dysfunctional and unresponsive to the signal. Both failure modes are hypotheses derived from mechanistic evidence; neither has been directly demonstrated in critically ill patients.

Current nutrition guidance uses recovery of enteral tolerance as a pragmatic milestone for reducing intravenous supplementation. However, feeding tolerance does not directly measure micronutrient absorptive capacity. Future trials should therefore test whether gastrointestinal phenotype modifies the relative biological effects of enteral and intravenous micronutrient delivery, rather than using citrulline or I-FABP as unvalidated bedside thresholds for route selection.

### 3.7. Clinical Implications and Future Perspectives

#### 3.7.1. Implications for Clinical Practice

**Figure 2:**
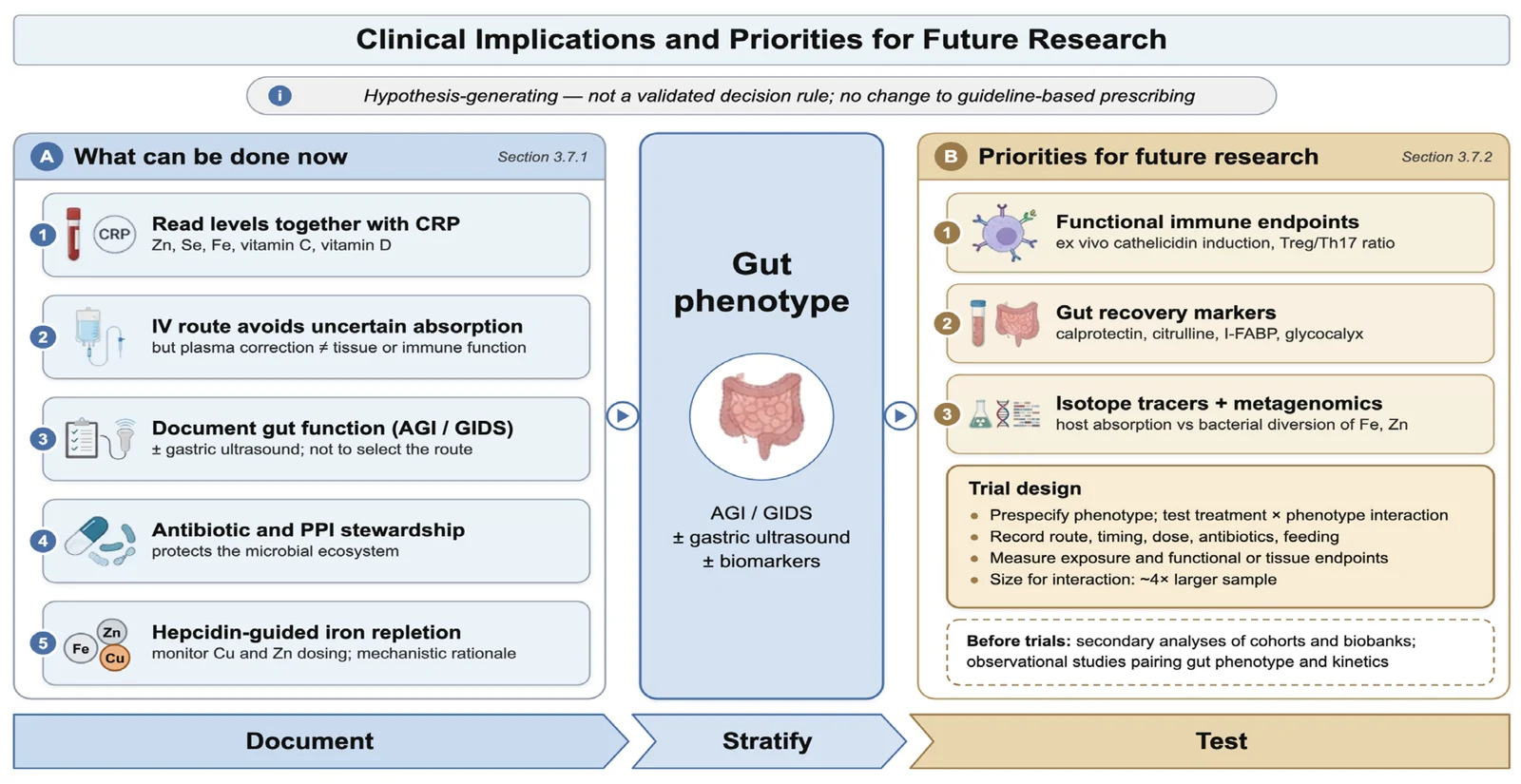
Clinical implications and priorities for future research. (**A**) What can be done now (Section 3.7.1): interpretation of circulating micronutrient concentrations together with C-reactive protein; intravenous administration avoids the uncertain absorption of enteral supplements, but correcting plasma levels is not equivalent to restoring tissue or immune function; documentation of gut function with AGI grade or GIDS (with or without gastric ultrasound), not to select the route of delivery; antibiotic and PPI stewardship; hepcidin-guided iron repletion with coordinated monitoring of copper and zinc dosing, supported by mechanistic evidence. (**B**) Priorities for future research (Section 3.7.2): functional immune endpoints, markers of gut recovery, and isotope-tracer studies combined with metagenomics; trials should prespecify gut phenotype as a stratification variable, test the treatment-by-phenotype interaction, record route, timing, dose, and antibiotic and feeding exposure, measure effective exposure and functional or tissue-level endpoints, and be sized for interaction; secondary analyses of existing cohorts and biobanks and observational studies should precede such trials. The framework is hypothesis-generating: none of its components has been validated as a clinical decision rule, and no change to guideline-based micronutrient prescribing is proposed. The strategies shown in Figure 1C are candidate strategies that require prospective evaluation (Section 5). Abbreviations: AGI, acute gastrointestinal injury; CRP, C-reactive protein; GIDS, Gastrointestinal Dysfunction Score; I-FABP, intestinal fatty acid-binding protein; IV, intravenous; PPI, proton-pump inhibitor; Th17, T helper 17 cell; Treg, regulatory T cell.

The framework proposed here is hypothesis-generating, and none of its components has been validated as a clinical decision rule; the evidence reviewed does not justify changing guideline-based micronutrient prescribing. Several practical implications can nevertheless be drawn from the better-established parts of the evidence. First, circulating concentrations of zinc, selenium, iron, vitamin C, and vitamin D should be interpreted together with an inflammatory marker such as C-reactive protein, because inflammation alone lowers these values (Section 3.1.1 and Section 3.3.3). Second, when the aim is to restore systemic availability, the intravenous route avoids the uncertain absorption of enteral supplements during severe gastrointestinal dysfunction, but correction of plasma levels should not be equated with restored tissue or immune function (Section 3.1.2 and Section 3.6.3); the harm signal reported with high-dose intravenous vitamin C in LOVIT (Table 4) is a reason for caution outside clinical trials. Third, routine bedside grading of gastrointestinal dysfunction (AGI grade or GIDS), with gastric ultrasound where available, can document gut function prospectively from routine data and would allow gut-stratified analyses of future trials (Section 3.6.1), although it should not be used to select the route of micronutrient delivery (Table 3). Fourth, antibiotic and proton-pump-inhibitor stewardship protects the microbial ecosystem and is justified independently of micronutrient considerations (Section 3.6.2). Finally, hepcidin-guided rather than indiscriminate iron repletion, and coordinated monitoring of copper and zinc dosing (Section 3.4.4), are consistent with the mechanistic evidence reviewed here. Figure 2 summarizes these practical implications together with the research priorities discussed in Section 3.7.2.

#### 3.7.2. Priorities for Future Research

Three concrete, testable extensions of this framework merit prioritization in future translational work. First, trials should adopt intracellular or functional immune readouts as secondary endpoints, for example, whole-blood ex vivo cathelicidin induction as a functional proxy for VDR signaling, or Treg/Th17 ratio in mucosal or peripheral samples, rather than relying solely on circulating micronutrient concentration, directly addressing the surrogate endpoint problem described in Section 3.5. Second, serial fecal calprotectin, borrowed from its established role in inflammatory bowel disease, warrants prospective validation as a noninvasive companion to citrulline and I-FABP for staging and tracking gut recovery in the ICU. Glycocalyx degradation markers should also be considered, as vascular endothelial damage could play a significant role in gut failure. Third, mechanistic studies should directly quantify the fraction of enterally administered iron and zinc that is diverted into the dysbiotic bacterial community versus absorbed by the host, using stable isotope tracer methodology combined with metagenomic profiling—a direct test of the bacterial sequestration hypothesis raised in Section 3.5, and a possible explanation for why enteral trace element repletion often fails to normalize host status despite adequate intake.

As a result, trials addressing the hypothesis that gut dysfunction modifies the response to micronutrients should (i) prespecify gut phenotype (AGI grade or GIDS, with or without gastric ultrasound and research biomarkers) as a stratification variable and test the treatment-by-phenotype interaction, rather than rely on post hoc subgroup analyses; (ii) record the route, timing, and dose of micronutrient delivery together with concomitant antibiotic and feeding exposure; (iii) include measures of effective exposure (for example plasma kinetics after a standardized enteral dose, or isotope-based absorption studies) and functional or tissue-level endpoints; and (iv) be sized for interaction effects, because a trial with 80% power for an overall effect had only 29% power to detect an interaction of the same magnitude, and roughly fourfold larger samples are needed to detect it with the same power [207]. Phenotype-based enrichment has been proposed as a way to address heterogeneity of treatment effect in sepsis [208]. Before such trials, secondary analyses of existing trial cohorts and biobanks, and prospective observational studies pairing gut phenotyping with micronutrient kinetics, could provide preliminary evidence on whether the hypothesis deserves testing.

## 4. Limitations

This review has several limitations that should be considered when interpreting its conclusions. First, it is a structured narrative review. Although the search covered three databases, applied explicit eligibility criteria, and used independent duplicate screening with arbitration, no protocol was registered, the search was not designed to capture every study on every micronutrient, and the synthesis is qualitative; selection bias and the authors’ interpretation may therefore have influenced the evidence presented (Section 2). Second, no formal risk-of-bias assessment or meta-analytic pooling was performed. Study quality was handled through an explicit evidence hierarchy rather than a validated tool, and the certainty of individual findings was not graded. Third, the evidence base is heterogeneous. It spans randomized trials, observational ICU cohorts, human studies outside the ICU, and animal and in vitro experiments, in adults and children, across different micronutrients, routes, doses, timings, definitions of gut dysfunction, and biomarkers; 104 of the 202 included records (51%) are narrative or expert reviews, and many mechanisms were characterized in non-ICU or preclinical settings, so their transferability to critically ill patients is uncertain (Supplementary Table S1).

Fourth, and most importantly, no randomized trial or well-designed observational study identified by our search has directly tested whether gastrointestinal dysfunction or microbiome state modifies the clinical response to micronutrient supplementation. The central proposition of this review, that gut failure may act as an unmeasured effect modifier, is therefore a hypothesis. The negative trials discussed in Section 3.5 may equally reflect timing, dose, population selection, the absence of true deficiency, or a lack of efficacy of the interventions, and several of the major trials used the intravenous route, for which impaired enteral absorption cannot be invoked. Fifth, the available gut biomarkers lack validation as decision tools (Table 3), and most of those discussed are research instruments. Sixth, the search ended on 30 September 2026, only English-language full texts were considered, and publication bias favoring positive mechanistic findings cannot be excluded; because recent publications were preferentially cited (Section 2.3), newer narrative syntheses may be over-represented relative to primary studies. Finally, the clinical implications in Section 3.7 are provisional and are not intended as practice recommendations.

## 5. Conclusions

Micronutrient supplementation trials in critical care have often failed to demonstrate consistent clinical benefit despite strong biological plausibility and frequent abnormalities in circulating micronutrient concentrations.

Observational data frequently associate low circulating micronutrient concentrations with adverse outcomes, although these concentrations are strongly influenced by inflammation, and the mechanistic literature provides a plausible biological rationale. A possible, but currently untested, contributor to the discordance between these associations and the neutral or negative results of supplementation trials is that the trials were designed, conducted, and analyzed without characterizing gut function, treating the gut as a passive conduit rather than a potentially injured organ. This explanation is neither the only one nor demonstrated: dose, timing, patient selection, the validity of plasma concentrations as a marker of deficiency, and the intrinsic efficacy of the interventions are alternative or additive explanations, and several major trials used the intravenous route, for which impaired absorption cannot account for the findings.

Gut failure, encompassing barrier disruption, epithelial atrophy, glycocalyx damage, transporter dysfunction, bile acid dysmetabolism, and microbiome dysbiosis, may disrupt micronutrient homeostasis at the absorptive, metabolic, and immunological levels, but the extent to which it does so in critically ill patients, and whether this alters the response to supplementation, has not been directly tested. Whether incorporating gut phenotyping into trial design would reduce heterogeneity of treatment effect and clarify the reasons for previous negative results is a testable hypothesis. Candidate strategies, each requiring prospective evaluation, include gut integrity stratification, microbiome phenotyping, phase-adapted timing and route selection, and microbiome-restoration protocols.

Future micronutrient trials should incorporate gastrointestinal phenotyping, route-specific exposure assessment, and functional or tissue-level endpoints to determine whether gut dysfunction truly modifies treatment response. This would directly test, rather than assume, the proposed gut–micronutrient–immune interaction. Until such evidence is available, gut failure should be regarded as a plausible but insufficiently tested effect modifier, not as an established explanation for the failure of micronutrient trials.

## Figures and Tables

**Table 3:** Potential tools for gastrointestinal phenotyping and gut-integrity assessment in critically ill patients: This table summarizes clinical, imaging, and circulating biomarkers that may contribute to gastrointestinal phenotyping in critically ill patients, highlighting their principal biological or clinical domain, available ICU evidence, limitations, and potential role in evaluating gut dysfunction and its relationship with micronutrient handling. These tools should be considered complementary rather than interchangeable, as no single biomarker currently provides a comprehensive measure of intestinal absorption, epithelial integrity, permeability, or tissue-level function.

Tool/ Biomarker	What It Mainly Reflects	Evidence in Critically Ill Patients	Major Limitations	Suggested Role in This Review	Representative References
Clinical GI phenotype: AGI grade/GIDS	Clinical severity of gastrointestinal dysfunction based on bedside signs and organ-support context	AGI provides a consensus clinical framework; GIDS was developed prospectively in a multicenter ICU cohort and adds prognostic information to general severity assessment	Does not directly measure enterocyte mass, permeability, or micronutrient absorption	Use as the primary clinical phenotyping layer in future trials, not as a direct marker of micronutrient absorptive capacity	[89,110]
Gastric ultrasound	Gastric content and antral dimensions; indirect information on upper-GI motility/emptying	Feasible at the bedside; in a pilot prospective ICU cohort, antral ultrasound measurements were associated with gastrointestinal dysfunction whereas citrulline and I-FABP had limited discriminatory value	Operator dependence; assesses mainly the stomach; does not measure small-bowel absorption or mucosal immune function	Useful adjunct to clinical phenotyping; not a surrogate for micronutrient absorption	[111]
Plasma citrulline	Functional enterocyte mass/small-bowel enterocyte metabolism	Low concentrations have been associated with illness severity and mortality; some studies report associations with AGI severity	Renal function and systemic metabolic factors affect levels; serial relationship with clinical GI dysfunction is inconsistent; no validated threshold for route selection	Prognostic/mechanistic research marker; should not be used to define recovery of enteral micronutrient absorption	[19,27,94,111,112]
I-FABP	Acute enterocyte injury and membrane damage	Elevated levels have been associated with shock severity and mortality in ICU cohorts	Short biological half-life; renal clearance and ischemic context may confound interpretation; limited performance as a serial functional marker	Marker of epithelial injury in research settings, not a stand-alone clinical measure of GI function	[27,94,96,111]
LBP	Systemic host response to lipopolysaccharide exposure/possible bacterial translocation	Biologically plausible marker of endotoxin exposure and translocation, studied in sepsis and barrier dysfunction	Not gut-specific; acute-phase response strongly affects concentrations; no validated ICU threshold linked to micronutrient handling	Exploratory translocation marker only	[113]
Zonulin	Proposed regulation of paracellular tight-junction permeability	Frequently used in clinical studies as a permeability-related readout	Commercial assays have major specificity problems and may detect proteins other than pre-haptoglobin 2; ICU validation is insufficient	Do not present as a validated permeability biomarker; preferably omit from any proposed clinical panel	[102,114]
Fecal calprotectin	Neutrophil-associated intestinal inflammation	Established in inflammatory bowel disease; preliminary signals exist in selected critically ill populations (Section 3.6.1)	ICU diarrhea, infection, antibiotics, feeding, and sampling logistics are major confounders; little validation for general ICU gut failure	Hypothesis-generating research adjunct only	[115,116,117]

Abbreviations: AGI, acute gastrointestinal injury; GI, gastrointestinal; GIDS, Gastrointestinal Dysfunction Score; I-FABP, intestinal fatty acid-binding protein; ICU, intensive care unit; LBP, lipopolysaccharide-binding protein.

**Table 4:** Landmark ICU supplementation trials relevant to the present review. All trials were randomized controlled trials in critically ill adults (direct ICU evidence).

Study	Population/*N*	Intervention and Route	Comparator	Primary Outcome	Main Finding	Gut/ Microbiome Assessment	Relevance to the Present Hypothesis
REDOXS, Heyland et al., 2013 [198]	Critically ill adults with multiorgan failure requiring mechanical ventilation; *N* = 1223	Glutamine and/or antioxidant supplementation in a 2 × 2 factorial design; supplements delivered enterally and parenterally	Placebo/no corresponding supplement	28-day mortality	Glutamine was associated with higher mortality (28-day trend, *p* = 0.05; significant in hospital and at 6 months); antioxidant supplementation did not improve mortality	No gastrointestinal phenotype, absorptive marker, or microbiome stratification	Mixed enteral/parenteral delivery makes simple malabsorption an insufficient explanation; timing, dose, systemic exposure, and tissue-level response remain relevant.
VITdAL-ICU, Amrein et al., 2014 [60]	Critically ill adults with 25(OH)D ≤ 20 ng/mL; *N* = 492 randomized (475 analyzed)	Vitamin D3 540,000 IU orally or via nasogastric tube, followed by 90,000 IU monthly for 5 months	Placebo	Hospital length of stay	No significant reduction in hospital length of stay or overall mortality; lower hospital mortality in the predefined severe-deficiency subgroup was hypothesis-generating	No assessment of gut function or microbiome	Suggests that baseline biological phenotype may modify response, but intestinal absorptive capacity was not characterized.
CITRIS-ALI, Fowler et al., 2019 [49]	Sepsis with ARDS; *N* = 167	Intravenous vitamin C 50 mg/kg every 6 h for 96 h	Placebo	Change in modified SOFA score and plasma CRP/thrombomodulin	No significant improvement in the prespecified primary organ-failure or biomarker outcomes	No gut-function or microbiome assessment	An intravenous trial: failure of the primary outcomes cannot be attributed to impaired enteral absorption.
VIOLET, PETAL Network, 2019 [59]	Critically ill patients screened as vitamin D deficient; *N* = 1360 randomized, 1078 with confirmed deficiency in the primary analysis	Single enteral dose of vitamin D3 540,000 IU	Placebo	90-day all-cause mortality	Marked biochemical correction of 25(OH)D without improvement in 90-day mortality	No gut-function or microbiome assessment	Direct example of biochemical correction without clinical benefit; whether gut phenotype modified biological response was not tested.
VITAMINS, Fujii et al., 2020 [48]	Septic shock; *N* = 216	IV vitamin C 1.5 g every 6 h + IV thiamine 200 mg every 12 h + IV hydrocortisone 50 mg every 6 h	IV hydrocortisone alone	Time alive and free of vasopressors through day 7	No significant increase in time alive and vasopressor-free	No gut-function or microbiome assessment	Combined intervention and intravenous micronutrient delivery; bypassing intestinal absorption does not guarantee clinical efficacy.
LOVIT, Lamontagne et al., 2022 [50]	Adults with sepsis receiving vasopressors in the ICU; *N* = 872 randomized (863 primary analysis)	IV vitamin C 50 mg/kg every 6 h for up to 96 h	Placebo	Death or persistent organ dysfunction at day 28	Primary outcome occurred more often with vitamin C (44.5% vs. 38.5%; RR 1.21, 95% CI 1.04–1.40)	No gut-function or microbiome assessment	Possible harm with high-dose systemic supplementation strongly argues against presenting gut failure as the sole explanation for negative micronutrient trials.

Abbreviations: ARDS, acute respiratory distress syndrome; CI, confidence interval; CITRIS-ALI, Vitamin C Infusion for Treatment in Sepsis-Induced Acute Lung Injury; CRP, C-reactive protein; ICU, intensive care unit; IU, international units; IV, intravenous; LOVIT, Lessening Organ Dysfunction with VITamin C; *N*, number of patients; PETAL, Prevention and Early Treatment of Acute Lung Injury; REDOXS, REducing Deaths due to OXidative Stress; RR, relative risk; SOFA, Sequential Organ Failure Assessment; VIOLET, Vitamin D to Improve Outcomes by Leveraging Early Treatment; VITAMINS, Vitamin C, Thiamine and Steroids in Sepsis; VITdAL-ICU, trial acronym as used by the original investigators; 25(OH)D, 25-hydroxyvitamin D.

## Data Availability

No new data were created or analyzed in this study. Data sharing is not applicable to this article.

## Supplementary Materials

The following supporting information can be downloaded at: https://www.mdpi.com/article/10.3390/nu18213516/s1, Table S1: Evidence map of the 202 records included in the narrative synthesis (study design, population or model, evidence tier, and manuscript sections in which each record is cited).

## Abbreviations

The following abbreviations are used in this manuscript:

AGI	Acute Gastrointestinal Injury
APACHE II	Acute Physiology and Chronic Health Evaluation II
ASBT	Apical sodium-dependent bile acid transporter
CD103	Cluster of differentiation 103
CI	Confidence interval
CRP	C-reactive protein
CRRT	Continuous renal replacement therapy
CYP27B1	Cytochrome P450 family 27 subfamily B member 1 (1α-hydroxylase)
DMT1	Divalent metal transporter 1
EN	Enteral nutrition
ESICM	European Society of Intensive Care Medicine
ESPEN	European Society for Clinical Nutrition and Metabolism
Foxp3	Forkhead box P3
FXR	Farnesoid X receptor
GALT	Gut-associated lymphoid tissue
GI	Gastrointestinal
GIDS	Gastrointestinal Dysfunction Score
GPR43	G protein-coupled receptor 43
GPR109A	G protein-coupled receptor 109A
HDAC	Histone deacetylase
HIF-1	Hypoxia-inducible factor 1
HLA-DR	Human leukocyte antigen-DR
HR	Hazard ratio
I-FABP	Intestinal fatty acid-binding protein
ICU	Intensive care unit
IF	Intestinal failure
IgA	Immunoglobulin A
IL	Interleukin
IV	Intravenous
L-FABP	Liver fatty acid-binding protein
LBP	Lipopolysaccharide-binding protein
LL-37	Cathelicidin antimicrobial peptide LL-37
LPS	Lipopolysaccharide
MODS	Multiple organ dysfunction syndrome
MUC2	Mucin 2
NF-κB	Nuclear factor kappa B
NLRP3	NLR family pyrin domain-containing protein 3
PRISMA	Preferred Reporting Items for Systematic reviews and Meta-Analyses
RA	All-trans retinoic acid
RALDH	Retinaldehyde dehydrogenase
RAR	Retinoic acid receptor
RCT	Randomized controlled trial
ROS	Reactive oxygen species
RR	Relative risk
RXR	Retinoid X receptor
SANRA	Scale for the Assessment of Narrative Review Articles
SCFA	Short-chain fatty acid
SOFA	Sequential Organ Failure Assessment
SVCT	Sodium-dependent vitamin C transporter
TGF-β	Transforming growth factor beta
TGR5	Takeda G protein-coupled receptor 5 (GPBAR1)
Th17	T helper 17 cell
TLR4	Toll-like receptor 4
TMAO	Trimethylamine *N*-oxide
TNF-α	Tumor necrosis factor alpha
Treg	Regulatory T cell
VAD	Vitamin A deficiency
VAP	Ventilator-associated pneumonia
VDR	Vitamin D receptor
ZIP	Zrt/Irt-related protein (SLC39 zinc importer family)
ZnT	Zinc transporter (SLC30 family)
ZO-1	Zonula occludens-1
25(OH)D	25-hydroxyvitamin D
1,25(OH)_2_D	1,25-dihydroxyvitamin D

## Appendix A

PubMed/MEDLINE search string:

(“Micronutrients”[MeSH Terms] OR “micronutrient*”[tiab] OR “Trace Elements”[MeSH Terms] OR “trace element*”[tiab] OR “Ascorbic Acid”[MeSH Terms] OR “vitamin C”[tiab] OR “Vitamin D”[MeSH Terms] OR “vitamin D”[tiab] OR “Vitamin A”[MeSH Terms] OR “retino*”[tiab] OR “Zinc”[MeSH Terms] OR “zinc”[tiab] OR “Selenium”[MeSH Terms] OR “selenium”[tiab] OR “Thiamine”[MeSH Terms] OR “thiamine”[tiab] OR “Copper”[MeSH Terms] OR “copper”[tiab] OR “Iron”[MeSH Terms] OR “iron”[tiab]) AND (“Critical Illness”[MeSH Terms] OR “critical* ill*”[tiab] OR “Sepsis”[MeSH Terms] OR “sepsis”[tiab] OR “septic shock”[tiab] OR “Intensive Care Units”[MeSH Terms] OR “intensive care”[tiab] OR “ICU”[tiab] OR “Multiple Organ Failure”[MeSH Terms] OR “multiorgan failure”[tiab]) AND (“Gastrointestinal Diseases”[MeSH Terms] OR “gut failure”[tiab] OR “acute gastrointestinal injury”[tiab] OR “Short Bowel Syndrome”[MeSH Terms] OR “intestinal failure”[tiab] OR “Permeability”[MeSH Terms] OR “intestinal permeability”[tiab] OR “gut barrier”[tiab] OR “Dysbiosis”[MeSH Terms] OR “dysbiosis”[tiab] OR “Gastrointestinal Microbiome”[MeSH Terms] OR “microbiot*”[tiab] OR “microbiom*”[tiab] OR “Bile Acids and Salts”[MeSH Terms] OR “bile acid*”[tiab] OR “Fatty Acids, Volatile”[MeSH Terms] OR “short-chain fatty acid*”[tiab] OR “Bacterial Translocation”[MeSH Terms] OR “bacterial translocation”[tiab] OR “Lymphoid Tissue”[MeSH Terms] OR “GALT”[tiab] OR “Homeostasis”[MeSH Terms] OR “immune homeostasis”[tiab] OR “mucosal immunity”[tiab]) AND (“1990/01/01”[PDAT]: “2026/09/30”[PDAT])

Embase search string (Emtree/free text):

(‘micronutrient’/exp OR ‘trace element’/exp OR ‘ascorbic acid’/exp OR ‘vitamin D’/exp OR ‘retinol’/exp OR ‘zinc’/exp OR ‘selenium’/exp OR ‘thiamine’/exp OR ‘copper’/exp OR ‘iron’/exp) AND (‘critical illness’/exp OR ‘sepsis’/exp OR ‘intensive care unit’/exp OR ‘multiple organ failure’/exp) AND (‘intestine failure’/exp OR ‘intestine permeability’/exp OR ‘dysbiosis’/exp OR ‘intestine flora’/exp OR ‘bile acid’/exp OR ‘short chain fatty acid’/exp OR ‘bacterial translocation’/exp OR ‘gut associated lymphoid tissue’/exp OR ‘mucosal immunity’/exp OR ‘gut failure’:ti,ab OR ‘acute gastrointestinal injury’:ti,ab OR ‘gut barrier’:ti,ab) AND [1990–2026]/py

CENTRAL search string:

(micronutrient* OR “trace element*” OR “vitamin C” OR “ascorbic acid” OR “vitamin D” OR “vitamin A” OR retino* OR zinc OR selenium OR thiamine OR copper OR iron):ti,ab,kw AND (“critical* ill*” OR sepsis OR “septic shock” OR “intensive care” OR ICU OR “organ failure”):ti,ab,kw AND (“gut failure” OR “acute gastrointestinal injury” OR “intestinal failure” OR “intestinal permeability” OR “gut barrier” OR dysbiosis OR microbiot* OR microbiom* OR “bile acid*” OR “short-chain fatty acid*” OR “bacterial translocation” OR “mucosal immunity”):ti,ab,kw, Publication Year from 1990 to 2026.
