# Supplementary material for: Gut Failure as a Potential Unmeasured Modifier of Micronutrient Response in Critical Illness: A Structured Narrative Review of Absorption, Metabolism, and Immune Function
Source: Nutrients. 2026 Oct 26;18(21):3516. doi: 10.3390/nu18213516 (PMC13765264; doi:10.3390/nu18213516)
Supplement: Supplementary file 1 [file nutrients-18-03516-s001.zip › nutrients-4606087-supplementary.pdf]

Supplementary Table S1. Evidence map of the 202 records included in the narrative synthesis

Gut Failure as a Potential Unmeasured Modifier of Micronutrient Response in Critical Illness: A Structured Narrative Review of Absorption, Metabolism, and Immune Function

This table maps each of the 202 records cited in the manuscript (reference numbers as in the reference list of the manuscript) to its study type, to the population or model on which its evidence rests, and to the manuscript sections in which it is used. It supports the statements on the strength of evidence in Section 2.3, Section 3, and Table 1. Six further references (28, 31, 35, 36, 207, and 208), added during revision, are not part of the 202 records identified by the search and are not listed here.

Evidence tier. Tier 1, critically ill patients (direct ICU evidence); tier 2, human studies outside the ICU or mixed human and mechanistic evidence (indirect evidence); tier 3, animal or in vitro studies (preclinical evidence). For secondary sources (guidelines, systematic reviews, narrative reviews) the tier reflects the population on which the source mainly reports; reviews that integrate human and preclinical mechanisms were assigned to tier 2. The tiers describe the type and setting of the evidence, not its methodological quality; no formal risk-of-bias assessment was performed.

Cited in. Section numbers refer to the manuscript (1 = Introduction); “Table n” indicates a citation within a table or its caption; “Fig 1 caption” indicates a citation in the legend of Figure 1.

Summary by study type and evidence tier (number of records)

Study type	Tier 1	Tier 2	Tier 3	Total
Randomized controlled trial	14	1	–	15
Observational study	23	10	–	33
Systematic review ± meta-analysis	5	5	–	10
Guideline / consensus statement	3	3	–	6
Narrative / expert review	27	76	1	104
Editorial	1	–	–	1
Study protocol	1	–	–	1
Pharmacovigilance analysis + review	–	1	–	1
Animal study	–	–	12	12
In vitro / cell-based study	–	–	10	10
Animal and in vitro study	–	–	4	4
Experimental study (mixed animal/human or human challenge)	–	3	–	3
Laboratory study	–	1	1	2
**Total**	**74**	**100**	**28**	**202**

Evidence map

No.	First author, year	Title (as indexed)	Study type	Population / model	Evidence tier	Cited in	Note
1	Berger 2024	ESPEN practical short micronutrient guideline	Guideline / consensus statement	Human, non-ICU or mixed clinical settings	2 – Human, non-ICU or mixed	1; 3.1.1	ESPEN guideline covering several clinical settings, including critically ill patients
2	Singer 2023	ESPEN practical and partially revised guideline: Clinical nutrition in the intensive care unit	Guideline / consensus statement	Critically ill adults	1 – Critically ill patients	1; 3.1.1	
3	Stoppe 2026	Micronutrients in adult critically ill patients: lessons learned and the path toward precision	Narrative / expert review	Critically ill adults	1 – Critically ill patients	1; 3.1.2	
4	Koekkoek 2021	Micronutrient deficiencies in critical illness	Observational study	Critically ill adults	1 – Critically ill patients	1; 3.1.1; 3.3.3; 3.4.4; 3.5; 3.6.2; Fig 1 caption	Prospective cohort
5	Gudivada 2021	Antioxidant micronutrient supplementation in critically ill adults: A systematic review with meta-analysis and trial sequential analysis	Systematic review ± meta-analysis	Critically ill adults	1 – Critically ill patients	1	
6	Gundogan 2022	Serum micronutrient levels in critically ill patients receiving continuous renal replacement therapy: A prospective, observational study	Observational study	Critically ill adults	1 – Critically ill patients	1	Patients on CRRT

No.	First author, year	Title (as indexed)	Study type	Population / model	Evidence tier	Cited in	Note
7	Adolph 2026	Current concepts on feeding the critically ill patient: a narrative review	Narrative / expert review	Critically ill adults	1 – Critically ill patients	1	
8	McMillan 2019	Relationship between nutritional status and the systemic inflammatory response: micronutrients	Systematic review ± meta-analysis	Human, non-ICU or mixed clinical settings	2 – Human, non-ICU or mixed	1; 3.3.3; Fig 1 caption	Acute (surgical) and chronic tissue injury
9	Berger 2020	Trace element and vitamin deficiency: quantum medicine or essential prescription?	Narrative / expert review	Critically ill adults	1 – Critically ill patients	1	
10	Hu 2020	Early Enteral Nutrition Preserves Intestinal Barrier Function through Reducing the Formation of Neutrophil Extracellular Traps (NETs) in Critically…	Observational study	Critically ill adults	1 – Critically ill patients	1	Intestinal biopsies, critically ill surgical patients
11	Schörghuber 2018	Effects of enteral nutrition on gastrointestinal function in patients who are critically ill	Narrative / expert review	Critically ill adults	1 – Critically ill patients	1; 3.2; 3.3.1; Fig 1 caption; Table 2	
12	Patel 2026	Nutrition Therapy in Critically Ill Adults	Narrative / expert review	Critically ill adults	1 – Critically ill patients	1; 3.1.2; 3.2; 3.3.1; 3.5; Fig 1 caption	
13	Soranno 2025	A review of gut failure as a cause and consequence of critical illness	Narrative / expert review	Critically ill (adults and children)	1 – Critically ill patients	1; 3.2; 3.3.1; Fig 1 caption	
14	Wozniak 2022	The central and biodynamic role of gut microbiota in critically ill patients	Narrative / expert review	Critically ill (adults and children)	1 – Critically ill patients	1	
15	Schlechte 2023	Dysbiosis of a microbiota-immune metasystem in critical illness is associated with nosocomial infections	Observational study	Critically ill adults	1 – Critically ill patients	1; 3.4.6; Fig 1 caption	ICU cohort with additional murine experiments
16	Obermüller 2020	Examination of intestinal ultrastructure, bowel wall apoptosis and tight junctions in the early phase of sepsis	Animal study	Animal model	3 – Preclinical	1	Murine model of sepsis
17	Lazzarin 2024	Editorial: Micronutrients and critically ill patients	Editorial	Critically ill adults	1 – Critically ill patients	1	
18	Mortillaro 1976	Interaction of capillary and tissue forces in the cat small intestine	Animal study	Animal model	3 – Preclinical	1	Cat small intestine, 1976
19	Piton 2010	Plasma citrulline kinetics and prognostic value in critically ill patients	Observational study	Critically ill adults	1 – Critically ill patients	1; Table 3	
20	Fagoni 2020	The IN-PANCIA Study: Clinical Evaluation of Gastrointestinal Dysfunction and Failure, Multiple Organ Failure, and Levels of Citrulline in Criticall…	Observational study	Critically ill adults	1 – Critically ill patients	1	
21	Gardner G. Micronutrient supplementation in patients with malabsorptive conditions. Nut in Clin Prac. 2024 Oct;39(5):1102–18. doi:10.1002/ncp.11190 2024	Micronutrient supplementation in patients with malabsorptive conditions	Narrative / expert review	Human, non-ICU or mixed clinical settings	2 – Human, non-ICU or mixed	1	Malabsorptive conditions
22	Berger 2020	Nutrition and Micronutrient Therapy in Critical Illness Should Be Individualized	Narrative / expert review	Critically ill adults	1 – Critically ill patients	1	
23	Berger 2021	Micronutrients early in critical illness, selective or generous, enteral or intravenous?	Narrative / expert review	Critically ill adults	1 – Critically ill patients	1; 3.3.3	

No.	First author, year	Title (as indexed)	Study type	Population / model	Evidence tier	Cited in	Note
24	Tomkins A. Assessing Micronutrient Status in the Presence of Inflammation. The Journal of Nutrition. 2003 May;133(5):1649S-1655S. doi:10.1093/jn/133.5.1649S 2003	Assessing micronutrient status in the presence of inflammation	Narrative / expert review	Human, non-ICU or mixed clinical settings	2 – Human, non-ICU or mixed	1	
25	Zheng 2026	The interplay between gastrointestinal dysfunction and gut microbiota dynamics in sepsis	Narrative / expert review	Critically ill adults	1 – Critically ill patients	1	Sepsis
26	Meng 2017	New insights into the gut as the driver of critical illness and organ failure	Narrative / expert review	Critically ill (adults and children)	1 – Critically ill patients	1	
27	Piton 2013	Enterocyte damage in critically ill patients is associated with shock condition and 28-day mortality	Observational study	Critically ill adults	1 – Critically ill patients	1; 3.6.1; Fig 1 caption; Table 3	
29	Zhang 2018	Prevalence and outcome of acute gastrointestinal injury in critically ill patients: A systematic review and meta-analysis	Systematic review ± meta-analysis	Critically ill adults	1 – Critically ill patients	3.2; 1	14 studies
30	Hu 2017	Severity of acute gastrointestinal injury grade is a predictor of all-cause mortality in critically ill patients: a multicenter, prospective, obser…	Observational study	Critically ill adults	1 – Critically ill patients	3.2; 1	Multicenter prospective, 550 patients
32	Blaauw 2026	Micronutrient trials in critical illness - new evidence since 2023	Narrative / expert review	Critically ill adults	1 – Critically ill patients	3.1.1; 3.1.2; 3.3.3; 3.5; Table 1; 1	
33	De Man 2025	What do we know about micronutrients in critically ill patients? A narrative review	Narrative / expert review	Critically ill adults	1 – Critically ill patients	Table 1; 1	
34	Corriero 2022	The central role of the gut in intensive care	Narrative / expert review	Critically ill adults	1 – Critically ill patients	3.3.4; 1	
37	Rozemeijer 2024	Micronutrient Status of Critically Ill Patients with COVID-19 Pneumonia	Observational study	Critically ill adults	1 – Critically ill patients	3.1.1	COVID-19 ICU cohort
38	Costa 2022	Insights Into Thiamine Supplementation in Patients With Septic Shock	Narrative / expert review	Critically ill adults	1 – Critically ill patients	3.1.1	Septic shock
39	Saglietti 2023	Role of Magnesium in the Intensive Care Unit and Immunomodulation: A Literature Review	Narrative / expert review	Critically ill adults	1 – Critically ill patients	3.1.1; Table 1	
40	Marelli 2026	Magnesium in Neurocritical Care: Clinical Relevance, Status Assessment, and Practical Implications for Outcomes-A Narrative Review	Narrative / expert review	Critically ill adults	1 – Critically ill patients	3.1.1; Table 1	Neurocritical care
41	Stoppe 2024	Micronutrients as therapy in critical illness	Narrative / expert review	Critically ill adults	1 – Critically ill patients	3.1.2	
42	Lu 2023	Ion channels and transporters regulate nutrient absorption in health and disease	Narrative / expert review	Human and preclinical (mechanistic)	2 – Human, non-ICU or mixed	3.1.2	
43	Seldén 2025	Net albumin leakage in patients in the ICU with suspected sepsis. A prospective analysis using mass balance calculations	Observational study	Critically ill adults	1 – Critically ill patients	3.1.2	Small physiological cohort (n = 10)
44	Gombart 2020	A Review of Micronutrients and the Immune System-Working in Harmony to Reduce the Risk of Infection	Narrative / expert review	Human and preclinical (mechanistic)	2 – Human, non-ICU or mixed	3.1.2	
45	De Medeiros 2018	Modulation of Intestinal Immune and Barrier Functions by Vitamin A: Implications for Current Understanding of Malnutrition and Enteric Infections i…	Narrative / expert review	Human and preclinical (mechanistic)	2 – Human, non-ICU or mixed	3.1.2	Paediatric malnutrition and enteric infection
46	Ohashi 2019	Contribution of Zinc and Zinc Transporters in the Pathogenesis of Inflammatory Bowel Diseases	Narrative / expert review	Human and preclinical (mechanistic)	2 – Human, non-ICU or mixed	3.1.2; 3.3.1; 3.4.1; Fig 1 caption; Table 1	Inflammatory bowel disease

No.	First author, year	Title (as indexed)	Study type	Population / model	Evidence tier	Cited in	Note
47	Wang 2022	Review article: insights into the bile acid-gut microbiota axis in intestinal failure-associated liver disease-redefining the treatment approach	Narrative / expert review	Human, non-ICU or mixed clinical settings	2 – Human, non-ICU or mixed	3.1.2; 3.3.4	Intestinal failure-associated liver disease
48	Fujii 2020	Effect of Vitamin C, Hydrocortisone, and Thiamine vs Hydrocortisone Alone on Time Alive and Free of Vasopressor Support Among Patients With Septic …	Randomized controlled trial	Critically ill adults	1 – Critically ill patients	Table 1; Table 4	Septic shock; combination regimen
49	Fowler AA 3rd 2019	Effect of Vitamin C Infusion on Organ Failure and Biomarkers of Inflammation and Vascular Injury in Patients With Sepsis and Severe Acute Respirato…	Randomized controlled trial	Critically ill adults	1 – Critically ill patients	Table 1; Table 4	Sepsis with ARDS; intravenous
50	Lamontagne 2022	Intravenous Vitamin C in Adults with Sepsis in the Intensive Care Unit	Randomized controlled trial	Critically ill adults	1 – Critically ill patients	Table 1; Table 4	Sepsis; intravenous
51	Reboul E. Proteins involved in fat-soluble vitamin and carotenoid transport across the intestinal cells: New insights from the past decade. Progress in Lipid Research. 2023 Jan;89:101208. doi:10.1016/j.plipres.2022.101208 2023	Proteins involved in fat-soluble vitamin and carotenoid transport across the intestinal cells: New insights from the past decade	Narrative / expert review	Human and preclinical (mechanistic)	2 – Human, non-ICU or mixed	3.3.1; Table 1	
52	Reboul 2012	Respective contributions of intestinal Niemann-Pick C1-like 1 and scavenger receptor class B type I to cholesterol and tocopherol uptake: in vivo v…	Animal and in vitro study	Animal and in vitro	3 – Preclinical	3.3.1; Table 1	
53	Chávez-Talavera 2017	Bile Acid Control of Metabolism and Inflammation in Obesity, Type 2 Diabetes, Dyslipidemia, and Nonalcoholic Fatty Liver Disease	Narrative / expert review	Human and preclinical (mechanistic)	2 – Human, non-ICU or mixed	3.3.1; 3.3.4; Table 1	Metabolic disease
54	Gonzalez 2016	An Intestinal Microbiota-Farnesoid X Receptor Axis Modulates Metabolic Disease	Narrative / expert review	Animal model	3 – Preclinical	3.3.1; Table 1	Review of mainly murine data
55	Abu-Wasel 2014	Liver disease secondary to intestinal failure	Narrative / expert review	Human, non-ICU or mixed clinical settings	2 – Human, non-ICU or mixed	3.3.1; Table 1	Intestinal failure
56	Kono 2015	Intracellular transport of fat-soluble vitamins A and E	Narrative / expert review	Human and preclinical (mechanistic)	2 – Human, non-ICU or mixed	3.3.1; Table 1	
57	Shen 2018	SR-B1: A Unique Multifunctional Receptor for Cholesterol Influx and Efflux	Narrative / expert review	Human and preclinical (mechanistic)	2 – Human, non-ICU or mixed	3.3.1; Table 1	
58	Damianos 2026	Mechanisms and pathophysiology leading to development of small intestinal microbial dysbiosis	Narrative / expert review	Human and preclinical (mechanistic)	2 – Human, non-ICU or mixed	3.3.1; Table 1	
59	PETAL Network 2019	Early High-Dose Vitamin D3 for Critically Ill, Vitamin D-Deficient Patients	Randomized controlled trial	Critically ill adults	1 – Critically ill patients	3.4.2; Table 1; Table 4	Enteral vitamin D3
60	Amrein 2014	Effect of high-dose vitamin D3 on hospital length of stay in critically ill patients with vitamin D deficiency: the VITdAL-ICU randomized clinical …	Randomized controlled trial	Critically ill adults	1 – Critically ill patients	Table 1; Table 4	Enteral vitamin D3
61	Desmarchelier 2018	Molecular interactions governing the incorporation of cholecalciferol and retinyl-palmitate in mixed taurocholate-lipid micelles	Laboratory study	In vitro / cell-based / laboratory	3 – Preclinical	3.3.1; Table 1	Micelle physical chemistry
62	Shale 2013	CD4(+) T-cell subsets in intestinal inflammation	Narrative / expert review	Human and preclinical (mechanistic)	2 – Human, non-ICU or mixed	3.4.3; Table 1	Intestinal inflammation
63	Cantorna 2019	Vitamin A and vitamin D regulate the microbial complexity, barrier function, and the mucosal immune responses to ensure intestinal homeostasis	Narrative / expert review	Human and preclinical (mechanistic)	2 – Human, non-ICU or mixed	3.4.3; Table 1	

No.	First author, year	Title (as indexed)	Study type	Population / model	Evidence tier	Cited in	Note
64	Camilleri M. Review: Human Intestinal Barrier—Optimal Measurement and Effects of Diet in the Absence of Overt Inflammation or Ulceration. Aliment Pharmacol Ther. 2025 Jul;62(2):128–45. doi:10.1111/apt.70225 2025	Review: Human Intestinal Barrier-Optimal Measurement and Effects of Diet in the Absence of Overt Inflammation or Ulceration	Narrative / expert review	Human, non-ICU or mixed clinical settings	2 – Human, non-ICU or mixed	3.4.3; 3.5; Table 1	
65	Feng 2020	Micronutrient deficiencies in pediatric short bowel syndrome: a 10-year review from an intestinal rehabilitation center in China	Observational study	Human, non-ICU or mixed clinical settings	2 – Human, non-ICU or mixed	3.4.3; 3.4.4; Table 1	Retrospective, paediatric short bowel syndrome
66	Donnino 2016	Randomized, Double-Blind, Placebo-Controlled Trial of Thiamine as a Metabolic Resuscitator in Septic Shock: A Pilot Study	Randomized controlled trial	Critically ill adults	1 – Critically ill patients	Table 1	Septic shock; pilot
67	Moskowitz 2023	Thiamine for Renal Protection in Septic Shock (TRPSS): A Randomized, Placebo-controlled, Clinical Trial	Randomized controlled trial	Critically ill adults	1 – Critically ill patients	Table 1	Septic shock
68	Sevransky 2021	Effect of Vitamin C, Thiamine, and Hydrocortisone on Ventilator- and Vasopressor-Free Days in Patients With Sepsis: The VICTAS Randomized Clinical …	Randomized controlled trial	Critically ill adults	1 – Critically ill patients	Table 1	Sepsis; combination regimen
69	Moskowitz 2020	Effect of Ascorbic Acid, Corticosteroids, and Thiamine on Organ Injury in Septic Shock: The ACTS Randomized Clinical Trial	Randomized controlled trial	Critically ill adults	1 – Critically ill patients	Table 1	Septic shock; combination regimen
70	Patel 2023	High-Dose IV Hydroxocobalamin (Vitamin B12) in Septic Shock: A Double-Blind, Allocation-Concealed, Placebo-Controlled Single-Center Pilot Randomize…	Randomized controlled trial	Critically ill adults	1 – Critically ill patients	Table 1	Septic shock; pilot, hydroxocobalamin
71	Bonaventura 2016	A Feedback Loop between Inflammation and Zn Uptake	In vitro / cell-based study	In vitro / cell-based / laboratory	3 – Preclinical	3.3.1; Fig 1 caption; Table 1	Synoviocyte model
72	Otsuka 2025	Zinc transporter dynamics in the pathogenesis of 2,4,6-trinitrobenzenesulfonic acid/ethanol-induced ileitis in mice	Animal study	Animal model	3 – Preclinical	3.3.1; Fig 1 caption; Table 1	Murine ileitis; authors missing in the reference list
73	Prasad 2011	Zinc-suppressed inflammatory cytokines by induction of A20-mediated inhibition of nuclear factor-κB	In vitro / cell-based study	In vitro / cell-based / laboratory	3 – Preclinical	3.4.1; Table 1	Cell lines
74	Mertens 2015	Low zinc and selenium concentrations in sepsis are associated with oxidative damage and inflammation	Observational study	Critically ill adults	1 – Critically ill patients	3.4.1; Table 1	Sepsis cohort
75	Sherlock 2020	Hepatic-Specific Decrease in the Expression of Selenoenzymes and Factors Essential for Selenium Processing After Endotoxemia	Animal study	Animal model	3 – Preclinical	3.3.3; Fig 1 caption; Table 1	Murine endotoxaemia
76	Renko 2009	Down-regulation of the hepatic selenoprotein biosynthesis machinery impairs selenium metabolism during the acute phase response in mice	Animal study	Animal model	3 – Preclinical	3.3.3; Fig 1 caption; Table 1	Murine acute-phase response
77	Silveira 2023	Assessment of trace elements in critically ill patients with systemic inflammatory response syndrome: A systematic review	Systematic review ± meta-analysis	Critically ill (adults and children)	1 – Critically ill patients	3.4.1; Table 1	Systemic inflammatory response syndrome
78	Kretz-Remy 2001	Selenium: a key element that controls NF-kappa B activation and I kappa B alpha half life	Narrative / expert review	Human and preclinical (mechanistic)	2 – Human, non-ICU or mixed	Table 1	Largely cell-based literature
79	Ganz T. Anemia of Inflammation. Longo 2019	Anemia of Inflammation	Narrative / expert review	Human, non-ICU or mixed clinical settings	2 – Human, non-ICU or mixed	3.3.3; Fig 1 caption; Table 1	
80	Stefanache 2023	Understanding How Minerals Contribute to Optimal Immune Function	Narrative / expert review	Human and preclinical (mechanistic)	2 – Human, non-ICU or mixed	3.4.4; Table 1	

No.	First author, year	Title (as indexed)	Study type	Population / model	Evidence tier	Cited in	Note
81	Bushman 2025	The exploitation of nutrient metals by bacteria for survival and infection in the gut	Narrative / expert review	Human and preclinical (mechanistic)	2 – Human, non-ICU or mixed	3.5; Table 1	
82	Nishito 2018	Absorption Mechanisms of Iron, Copper, and Zinc: An Overview	Narrative / expert review	Human and preclinical (mechanistic)	2 – Human, non-ICU or mixed	Table 1	
83	Alexander 2008	Molecular determinants of magnesium homeostasis: insights from human disease	Narrative / expert review	Human and preclinical (mechanistic)	2 – Human, non-ICU or mixed	Table 1	
84	Touyz 2024	Magnesium Disorders	Narrative / expert review	Human, non-ICU or mixed clinical settings	2 – Human, non-ICU or mixed	3.3.1; Fig 1 caption; Table 1	
85	Kröse 2024	Magnesium biology	Narrative / expert review	Human and preclinical (mechanistic)	2 – Human, non-ICU or mixed	3.3.1; Fig 1 caption; Table 1	
86	Hernando 2018	Mechanisms and Regulation of Intestinal Phosphate Absorption	Narrative / expert review	Human and preclinical (mechanistic)	2 – Human, non-ICU or mixed	Table 1	
87	Da Silva 2020	ASPEN Consensus Recommendations for Refeeding Syndrome	Guideline / consensus statement	Human, non-ICU or mixed clinical settings	2 – Human, non-ICU or mixed	Table 1	Refeeding syndrome consensus (not ICU-specific)
88	Schuetz 2021	Management of disease-related malnutrition for patients being treated in hospital	Narrative / expert review	Human, non-ICU or mixed clinical settings	2 – Human, non-ICU or mixed	Table 1	Hospital malnutrition
89	Reintam Blaser 2012	Gastrointestinal function in intensive care patients: terminology, definitions and management. Recommendations of the ESICM Working Group on Abdomi…	Guideline / consensus statement	Critically ill adults	1 – Critically ill patients	3.2; 3.6.1; Fig 1 caption; Table 2; Table 3	ESICM working group recommendations
90	Padar 2019	Gastrointestinal failure affects outcome of intensive care	Observational study	Critically ill adults	1 – Critically ill patients	3.2	
91	Li 2016	Association between acute gastrointestinal injury grading system and disease severity and prognosis in critically ill patients: A multicenter, pros…	Observational study	Critically ill adults	1 – Critically ill patients	3.2	
92	Berger 2022	Management of gastrointestinal failure in the adult critical care setting	Narrative / expert review	Critically ill adults	1 – Critically ill patients	3.2	
93	Pironi 2018	Intestinal failure in adults: Recommendations from the ESPEN expert groups	Guideline / consensus statement	Human, non-ICU or mixed clinical settings	2 – Human, non-ICU or mixed	3.2	ESPEN intestinal failure (not ICU-specific)
94	Tyszko 2023	Citrulline, Intestinal Fatty Acid-Binding Protein and the Acute Gastrointestinal Injury Score as Predictors of Gastrointestinal Failure in Patients…	Observational study	Critically ill adults	1 – Critically ill patients	3.2; Fig 1 caption; Table 2; Table 3	Sepsis and septic shock
95	Fishbein 2023	Antibiotic perturbations to the gut microbiome	Narrative / expert review	Human and preclinical (mechanistic)	2 – Human, non-ICU or mixed	3.2; Table 2	
96	Piton 2016	Biomarkers of gut barrier failure in the ICU	Narrative / expert review	Critically ill adults	1 – Critically ill patients	3.2; 3.6.1; Fig 1 caption; Table 2; Table 3	
97	Jozwiak 2022	Vasopressors and Risk of Acute Mesenteric Ischemia: A Worldwide Pharmacovigilance Analysis and Comprehensive Literature Review	Pharmacovigilance analysis + review	Human, non-ICU or mixed clinical settings	2 – Human, non-ICU or mixed	3.2; Table 2	Patients receiving vasopressors; not ICU-specific
98	Minerbi 2022	Gut Microbiome in Anesthesiology and Pain Medicine	Narrative / expert review	Human and preclinical (mechanistic)	2 – Human, non-ICU or mixed	3.2; Table 2	
99	Martinez 2023	Biomarkers and Functional Assays of Epithelial Barrier Disruption and Gastrointestinal Dysmotility in Critical Illness-A Narrative Review	Narrative / expert review	Critically ill (adults and children)	1 – Critically ill patients	3.2	
100	Neurath 2025	The intestinal barrier: a pivotal role in health, inflammation, and cancer	Narrative / expert review	Human and preclinical (mechanistic)	2 – Human, non-ICU or mixed	3.2	
101	Meyer 2023	Cytokines and intestinal epithelial permeability: A systematic review	Systematic review ± meta-analysis	Human and preclinical (mechanistic)	2 – Human, non-ICU or mixed	3.2	Mainly experimental permeability models
102	Donmez-Altuntas 2023	Gut barrier protein levels in serial blood samples from critically ill trauma patients during and after intensive care unit stay	Observational study	Critically ill adults	1 – Critically ill patients	3.2; Table 2; Table 3	Trauma ICU
103	Zhao 2016	PROGNOSTIC VALUE OF PLASMA TIGHT-JUNCTION PROTEINS FOR SEPSIS IN EMERGENCY DEPARTMENT: AN OBSERVATIONAL STUDY	Observational study	Human, non-ICU or mixed clinical settings	2 – Human, non-ICU or mixed	3.2; Table 2	Sepsis in the emergency department (not ICU)

No.	First author, year	Title (as indexed)	Study type	Population / model	Evidence tier	Cited in	Note
104	Eslamian 2019	Association of intestinal permeability with a NUTRIC score in critically ill patients	Observational study	Critically ill adults	1 – Critically ill patients	3.2	
105	Modi 2014	Antibiotics and the gut microbiota	Narrative / expert review	Human and preclinical (mechanistic)	2 – Human, non-ICU or mixed	Table 2	
106	Schwartz 2020	Understanding the impact of antibiotic perturbation on the human microbiome	Narrative / expert review	Human, non-ICU or mixed clinical settings	2 – Human, non-ICU or mixed	Table 2	
107	Taylor 2025	Antibiotics with anaerobic coverage are associated with disproportionate gut microbiota disruption in ICU patients: a multi-centre, longitudinal ob…	Observational study	Critically ill adults	1 – Critically ill patients	Table 2	Research letter, multicentre observational
108	Guyton 2017	The gut microbiota and gastrointestinal surgery	Narrative / expert review	Human and preclinical (mechanistic)	2 – Human, non-ICU or mixed	Table 2	Surgery
109	Khan 2021	Protective Treatments against Endothelial Glycocalyx Degradation in Surgery: A Systematic Review and Meta-Analysis	Systematic review ± meta-analysis	Human, non-ICU or mixed clinical settings	2 – Human, non-ICU or mixed	Table 2	Surgical patients (not ICU)
110	Reintam Blaser 2021	Development of the Gastrointestinal Dysfunction Score (GIDS) for critically ill patients - A prospective multicenter observational study (iSOFA study)	Observational study	Critically ill adults	1 – Critically ill patients	3.6.1; Fig 1 caption; Table 3	Multicenter prospective
111	Onuk 2023	Gastric ultrasound, citrulline, and intestinal fatty acid-binding protein as markers of gastrointestinal dysfunction in critically ill patients: A …	Observational study	Critically ill adults	1 – Critically ill patients	3.6.1; Fig 1 caption; Table 3	Pilot cohort
112	Blaser 2019	Citrulline and intestinal fatty acid-binding protein as biomarkers for gastrointestinal dysfunction in the critically ill	Narrative / expert review	Critically ill adults	1 – Critically ill patients	Table 3	
113	Seethaler 2021	Biomarkers for assessment of intestinal permeability in clinical practice	Observational study	Human, non-ICU or mixed clinical settings	2 – Human, non-ICU or mixed	3.6.1; Fig 1 caption; Table 3	Cross-sectional
114	Scheffler 2018	Widely Used Commercial ELISA Does Not Detect Precursor of Haptoglobin2, but Recognizes Properdin as a Potential Second Member of the Zonulin Family	Laboratory study	Human, non-ICU or mixed clinical settings	2 – Human, non-ICU or mixed	Table 3	Assay validation in a population cohort
115	Montalto 2013	Role of fecal calprotectin in gastrointestinal disorders	Narrative / expert review	Human, non-ICU or mixed clinical settings	2 – Human, non-ICU or mixed	3.6.1; Table 3	Incomplete entry in the reference list
116	Kudaş İ 2026	Calprotectin as a Novel Biomarker for Ischemic Colitis: Experimental Insights in a Rat Model	Animal study	Animal model	3 – Preclinical	3.6.1; Table 3	Rat model
117	Suttichaimongkol 2024	Diagnostic Performance of a Fecal Calprotectin Assay as a Biomarker for Mayo Endoscopic Subscore in Ulcerative Colitis: Result From a Tertiary Refe…	Observational study	Human, non-ICU or mixed clinical settings	2 – Human, non-ICU or mixed	Table 3	Ulcerative colitis
118	Li 2026	Diagnostic clues in patients with clinical malabsorption and pathological small intestinal villous atrophy: Immune-mediated type and beyond	Narrative / expert review	Human, non-ICU or mixed clinical settings	2 – Human, non-ICU or mixed	3.3.1; Fig 1 caption	
119	Duncan 2012	Quantitative data on the magnitude of the systemic inflammatory response and its effect on micronutrient status based on plasma measurements	Observational study	Human, non-ICU or mixed clinical settings	2 – Human, non-ICU or mixed	3.3.3; Fig 1 caption	Hospital laboratory database
120	Liuzzi 2005	Interleukin-6 regulates the zinc transporter Zip14 in liver and contributes to the hypozincemia of the acute-phase response	Animal and in vitro study	Animal and in vitro	3 – Preclinical	3.3.3; Fig 1 caption	
121	Liu 2025	Hypoferremic Response to Chronic Inflammation Is Controlled via the Hemojuvelin/Hepcidin/Ferroportin Axis and Does Not Involve Hepcidin-Independent…	Animal study	Animal model	3 – Preclinical	3.3.3; Fig 1 caption	
122	Hertli 2022	Molecular interactions between the intestinal microbiota and the host	Narrative / expert review	Human and preclinical (mechanistic)	2 – Human, non-ICU or mixed	3.3.2; 3.3.4; 3.4.5; Fig 1 caption	
123	Mann 2024	Short-chain fatty acids: linking diet, the microbiome and immunity	Narrative / expert review	Human and preclinical (mechanistic)	2 – Human, non-ICU or mixed	3.4.5; 3.5; Fig 1 caption	
124	Tan 2023	Dietary fiber and SCFAs in the regulation of mucosal immunity	Narrative / expert review	Human, non-ICU or mixed clinical settings	2 – Human, non-ICU or mixed	3.4.5; 3.5; Fig 1 caption	
125	Furusawa 2013	Commensal microbe-derived butyrate induces the differentiation of colonic regulatory T cells	Animal study	Animal model	3 – Preclinical	3.4.5; Fig 1 caption	Murine

No.	First author, year	Title (as indexed)	Study type	Population / model	Evidence tier	Cited in	Note
126	Clavel 2017	Deciphering interactions between the gut microbiota and the immune system via microbial cultivation and minimal microbiomes	Narrative / expert review	Human and preclinical (mechanistic)	2 – Human, non-ICU or mixed	3.4.5; Fig 1 caption	
127	Chang JT. Pathophysiology of Inflammatory Bowel Diseases. Longo 2020	Pathophysiology of Inflammatory Bowel Diseases	Narrative / expert review	Human, non-ICU or mixed clinical settings	2 – Human, non-ICU or mixed	3.4.5; Fig 1 caption	Inflammatory bowel disease
128	Weiner 2011	Oral tolerance	Narrative / expert review	Human and preclinical (mechanistic)	2 – Human, non-ICU or mixed	3.4.5; Fig 1 caption	
129	Neish AS. Microbes in Gastrointestinal Health and Disease. Gastroenterology. 2009 Jan;136(1):65–80. doi:10.1053/j.gastro.2008.10.080 2009	Microbes in gastrointestinal health and disease	Narrative / expert review	Human and preclinical (mechanistic)	2 – Human, non-ICU or mixed	3.4.5; Fig 1 caption	
130	Chen 2024	Gut microbiota and intestinal immunity-A crosstalk in irritable bowel syndrome	Narrative / expert review	Human, non-ICU or mixed clinical settings	2 – Human, non-ICU or mixed	3.4.5; Fig 1 caption	
131	Jirsova 2019	The Effect of Butyrate-Supplemented Parenteral Nutrition on Intestinal Defence Mechanisms and the Parenteral Nutrition-Induced Shift in the Gut Mic…	Animal study	Animal model	3 – Preclinical	3.4.5; Fig 1 caption	Rat model
132	Daniels 2026	The leaky gut and microbiome in critical illness: emerging insights into microbial "translocation"	Narrative / expert review	Critically ill adults	1 – Critically ill patients	3.4.6; Fig 1 caption	
133	Guarner 2003	Gut flora in health and disease	Narrative / expert review	Human, non-ICU or mixed clinical settings	2 – Human, non-ICU or mixed	3.4.6; Fig 1 caption	Incomplete entry in the reference list
134	Magnan 2023	Role of gut microbiota and bacterial translocation in acute intestinal injury and mortality in patients admitted in ICU for septic shock	Observational study	Critically ill adults	1 – Critically ill patients	3.4.6; Fig 1 caption	Septic shock cohort
135	Larsson 2022	Understanding the role of endotoxin tolerance in chronic inflammatory conditions and periodontal disease	Narrative / expert review	Human, non-ICU or mixed clinical settings	2 – Human, non-ICU or mixed	3.4.6; Fig 1 caption	
136	Weisheit 2020	Sustained Immunoparalysis in Endotoxin-Tolerized Monocytic Cells	In vitro / cell-based study	In vitro / cell-based / laboratory	3 – Preclinical	3.4.6; Fig 1 caption	Monocytic cell line
137	Grondman 2019	Frontline Science: Endotoxin-induced immunotolerance is associated with loss of monocyte metabolic plasticity and reduction of oxidative burst	Experimental study (mixed animal/human or human challenge)	Human, non-ICU or mixed clinical settings	2 – Human, non-ICU or mixed	3.4.6; Fig 1 caption	Human endotoxaemia model and ex vivo monocytes
138	Liu 2023	Association between Gastrointestinal Dysfunction Score (GIDS) and disease severity and prognosis in critically ill patients: A prospective, observa…	Observational study	Critically ill adults	1 – Critically ill patients	3.6.1; Fig 1 caption	Prospective
139	Sharif 2022	Probiotics in Critical Illness: A Systematic Review and Meta-Analysis of Randomized Controlled Trials	Systematic review ± meta-analysis	Critically ill (adults and children)	1 – Critically ill patients	3.6.2; Fig 1 caption	
140	Lee 2023	Benefits and harm of probiotics and synbiotics in adult critically ill patients. A systematic review and meta-analysis of randomized controlled tri…	Systematic review ± meta-analysis	Critically ill adults	1 – Critically ill patients	3.6.2; Fig 1 caption	
141	Johnstone 2021	Effect of Probiotics on Incident Ventilator-Associated Pneumonia in Critically Ill Patients: A Randomized Clinical Trial	Randomized controlled trial	Critically ill adults	1 – Critically ill patients	3.6.2; Fig 1 caption	PROSPECT
142	Prescott 2026	Surviving Sepsis Campaign: International Guidelines for Management of Sepsis and Septic Shock 2026	Guideline / consensus statement	Critically ill adults	1 – Critically ill patients	3.6.2; Fig 1 caption	Surviving Sepsis Campaign 2026
143	Wittekamp 2020	Selective decontamination of the digestive tract (SDD) in critically ill patients: a narrative review	Narrative / expert review	Critically ill adults	1 – Critically ill patients	3.6.2; Fig 1 caption	

No.	First author, year	Title (as indexed)	Study type	Population / model	Evidence tier	Cited in	Note
144	SuDDICU Investigators 2022	Effect of Selective Decontamination of the Digestive Tract on Hospital Mortality in Critically Ill Patients Receiving Mechanical Ventilation: A Ran…	Randomized controlled trial	Critically ill adults	1 – Critically ill patients	3.6.2; Fig 1 caption	
145	Saha 2019	Faecal microbiota transplantation for eradicating carriage of multidrug-resistant organisms: a systematic review	Systematic review ± meta-analysis	Human, non-ICU or mixed clinical settings	2 – Human, non-ICU or mixed	3.6.2; Fig 1 caption	Non-ICU populations
146	Cibulkova 2024	Allogenic faecal microbiota transplantation for antibiotic-associated diarrhoea in critically ill patients (FEBATRICE)-Study protocol for a multi-c…	Study protocol	Critically ill adults	1 – Critically ill patients	3.6.2; Fig 1 caption	Protocol only; no results
147	Barone 2022	Gut microbiome-micronutrient interaction: The key to controlling the bioavailability of minerals and vitamins?	Narrative / expert review	Human, non-ICU or mixed clinical settings	2 – Human, non-ICU or mixed	3.6.2; Fig 1 caption	
148	Hao 2021	Butyrate enhances CPT1A activity to promote fatty acid oxidation and iTreg differentiation	Animal and in vitro study	Animal and in vitro	3 – Preclinical	3.6.2; Fig 1 caption	
149	Serbanescu 2024	Impact of Fiber-Containing Enteral Nutrition on Microbial Community Dynamics in Critically Ill Trauma Patients: A Pilot-Randomized Trial	Randomized controlled trial	Critically ill adults	1 – Critically ill patients	3.6.2; Fig 1 caption	Pilot RCT;
150	McClave 2024	The importance of providing dietary fiber in medical and surgical critical care	Narrative / expert review	Critically ill adults	1 – Critically ill patients	3.6.2; Fig 1 caption	
151	Wei 2026	Gut dysbiosis‐derived butyrate loss predicts feeding intolerance: Multiomics evidence guiding nurse‐driven microbiota‐supportive interventions (Rev…	Narrative / expert review	Critically ill adults	1 – Critically ill patients	3.6.2; Fig 1 caption	
152	Fayasari 2026	Gut microbiota and iron deficiency anemia: Mechanisms, microbial signatures, and dietary interactions (A narrative review)	Narrative / expert review	Human, non-ICU or mixed clinical settings	2 – Human, non-ICU or mixed	3.6.2; Fig 1 caption	
153	Bushyhead 2022	Small Intestinal Bacterial Overgrowth-Pathophysiology and Its Implications for Definition and Management	Narrative / expert review	Human, non-ICU or mixed clinical settings	2 – Human, non-ICU or mixed	3.3.2	
154	Karl 2015	Fecal menaquinone profiles of overweight adults are associated with gut microbiota composition during a gut microbiota-targeted dietary intervention	Observational study	Human, non-ICU or mixed clinical settings	2 – Human, non-ICU or mixed	3.3.2	Dietary intervention, overweight adults
155	Karl 2017	Fecal concentrations of bacterially derived vitamin K forms are associated with gut microbiota composition but not plasma or fecal cytokine concent…	Randomized controlled trial	Human, non-ICU or mixed clinical settings	2 – Human, non-ICU or mixed	3.3.2	Secondary analysis of an RCT in healthy adults
156	Engevik 2019	Microbial Metabolic Capacity for Intestinal Folate Production and Modulation of Host Folate Receptors	In vitro / cell-based study	In vitro / cell-based / laboratory	3 – Preclinical	3.3.2	Bacterial and cell-based
157	Singh 2023	Butyrate producers, "The Sentinel of Gut": Their intestinal significance with and beyond butyrate, and prospective use as microbial therapeutics	Narrative / expert review	Human and preclinical (mechanistic)	2 – Human, non-ICU or mixed	3.3.2; 3.4.2	
158	Noble 2023	Defining Interactions Between the Genome, Epigenome, and the Environment in Inflammatory Bowel Disease: Progress and Prospects	Narrative / expert review	Human, non-ICU or mixed clinical settings	2 – Human, non-ICU or mixed	3.3.2	Inflammatory bowel disease
159	He 2025	Gut Microbiota-Derived Metabolites and Cardiovascular Disease: Focus on Trimethylamine-N-oxide	Narrative / expert review	Human and preclinical (mechanistic)	2 – Human, non-ICU or mixed	3.3.2	TMAO
160	Tang 2019	Intestinal Microbiota in Cardiovascular Health and Disease: JACC State-of-the-Art Review	Narrative / expert review	Human, non-ICU or mixed clinical settings	2 – Human, non-ICU or mixed	3.3.2	Cardiovascular
161	Sun 2016	Trimethylamine N-oxide induces inflammation and endothelial dysfunction in human umbilical vein endothelial cells via activating ROS-TXNIP-NLRP3 in…	In vitro / cell-based study	In vitro / cell-based / laboratory	3 – Preclinical	3.3.2	Endothelial cells
162	Zhu 2016	Gut Microbial Metabolite TMAO Enhances Platelet Hyperreactivity and Thrombosis Risk	Experimental study (mixed animal/human or human challenge)	Human and preclinical (mechanistic)	2 – Human, non-ICU or mixed	3.3.2	Murine thrombosis model and human data
163	Trøseid 2015	Microbiota-dependent metabolite trimethylamine-N-oxide is associated with disease severity and survival of patients with chronic heart failure	Observational study	Human, non-ICU or mixed clinical settings	2 – Human, non-ICU or mixed	3.3.2	Chronic heart failure

No.	First author, year	Title (as indexed)	Study type	Population / model	Evidence tier	Cited in	Note
164	Tang 2013	Intestinal microbial metabolism of phosphatidylcholine and cardiovascular risk	Experimental study (mixed animal/human or human challenge)	Human and preclinical (mechanistic)	2 – Human, non-ICU or mixed	3.3.2	Human cohort and murine experiments
165	Li 2017	Gut microbiota-dependent trimethylamine N-oxide in acute coronary syndromes: a prognostic marker for incident cardiovascular events beyond traditio…	Observational study	Human, non-ICU or mixed clinical settings	2 – Human, non-ICU or mixed	3.3.2	Acute coronary syndromes
166	Kullak-ublick 2004	Enterohepatic bile salt transporters in normal physiology and liver disease	Narrative / expert review	Human and preclinical (mechanistic)	2 – Human, non-ICU or mixed	3.3.4	
167	Simbrunner 2021	Review article: therapeutic aspects of bile acid signalling in the gut-liver axis	Narrative / expert review	Human, non-ICU or mixed clinical settings	2 – Human, non-ICU or mixed	3.3.4	
168	Schubert 2017	Interactions between bile salts, gut microbiota, and hepatic innate immunity	Narrative / expert review	Human and preclinical (mechanistic)	2 – Human, non-ICU or mixed	3.3.4	
169	Zhan 2020	Gut Microbiota-Bile Acid Crosstalk in Diarrhea-Irritable Bowel Syndrome	Narrative / expert review	Human, non-ICU or mixed clinical settings	2 – Human, non-ICU or mixed	3.3.4	
170	Yan 2025	Bile acid-mediated gut-liver axis crosstalk: the role of nuclear receptor signaling in dynamic regulation of inflammatory networks	Narrative / expert review	Human and preclinical (mechanistic)	2 – Human, non-ICU or mixed	3.3.4	
171	Kean 2022	Profiling gut microbiota and bile acid metabolism in critically ill children	Observational study	Critically ill children	1 – Critically ill patients	3.3.4	
172	Saeed 2017	The interrelationship between bile acid and vitamin A homeostasis	Narrative / expert review	Human and preclinical (mechanistic)	2 – Human, non-ICU or mixed	3.3.4	
173	Briassoulis 2023	The Anti-Oxidative, Anti-Inflammatory, Anti-Apoptotic, and Anti-Necroptotic Role of Zinc in COVID-19 and Sepsis	Narrative / expert review	Human and preclinical (mechanistic)	2 – Human, non-ICU or mixed	3.4.1	COVID-19 and sepsis
174	Hongxia 2019	Zinc inhibited LPS-induced inflammatory responses by upregulating A20 expression in microglia BV2 cells	In vitro / cell-based study	In vitro / cell-based / laboratory	3 – Preclinical	3.4.1	Microglial cell line
175	Niu 2022	Selenium metabolism and regulation of immune cells in immune-associated diseases	Narrative / expert review	Human, non-ICU or mixed clinical settings	2 – Human, non-ICU or mixed	3.4.1	
176	Prabhu 2002	Selenium deficiency increases the expression of inducible nitric oxide synthase in RAW 264.7 macrophages: role of nuclear factor-kappaB in up-regul…	In vitro / cell-based study	In vitro / cell-based / laboratory	3 – Preclinical	3.4.1	Macrophage cell line
177	Fakhoury HanaMA 2020	Vitamin D and intestinal homeostasis: Barrier, microbiota, and immune modulation	Narrative / expert review	Human and preclinical (mechanistic)	2 – Human, non-ICU or mixed	3.4.2	
178	Barbáchano 2017	The endocrine vitamin D system in the gut	Narrative / expert review	Human and preclinical (mechanistic)	2 – Human, non-ICU or mixed	3.4.2	
179	Wang 2004	Cutting Edge: 1,25-Dihydroxyvitamin D3 Is a Direct Inducer of Antimicrobial Peptide Gene Expression	In vitro / cell-based study	In vitro / cell-based / laboratory	3 – Preclinical	3.4.2	Incomplete entry in the reference list
180	Ismailova 2022	Vitamin D, infections and immunity	Narrative / expert review	Human, non-ICU or mixed clinical settings	2 – Human, non-ICU or mixed	3.4.2	
181	Zeb 2026	Effect of Vitamin D Supplementation on Human Gut Microbiota: A Systematic Review of Randomized Controlled Trials	Systematic review ± meta-analysis	Human, non-ICU or mixed clinical settings	2 – Human, non-ICU or mixed	3.4.2	RCTs in non-ICU populations
182	Zhang Y guo 2015	Tight junction CLDN2 gene is a direct target of the vitamin D receptor	Animal and in vitro study	Animal and in vitro	3 – Preclinical	3.4.2	
183	Kellermann 2020	Mucosal vitamin D signaling in inflammatory bowel disease	Narrative / expert review	Human, non-ICU or mixed clinical settings	2 – Human, non-ICU or mixed	3.4.2	Inflammatory bowel disease
184	Du 2017	Microbiota-Dependent Induction of Colonic Cyp27b1 Is Associated With Colonic Inflammation: Implications of Locally Produced 1,25-Dihydroxyvitamin D…	Animal study	Animal model	3 – Preclinical	3.4.2; 3.4.6	Murine colitis
185	Bora 2018	The Gut Microbiota Regulates Endocrine Vitamin D Metabolism through Fibroblast Growth Factor 23	Animal study	Animal model	3 – Preclinical	3.4.2	
186	Schoenmakers 2026	Alterations of vitamin D metabolism and requirements in clinical conditions with impaired gastro-intestinal and renal function and in critical illness	Narrative / expert review	Human and preclinical (mechanistic)	2 – Human, non-ICU or mixed	3.4.2	Includes critical illness

No.	First author, year	Title (as indexed)	Study type	Population / model	Evidence tier	Cited in	Note
187	Oliveira 2018	Impact of Retinoic Acid on Immune Cells and Inflammatory Diseases	Narrative / expert review	Human and preclinical (mechanistic)	2 – Human, non-ICU or mixed	3.4.3	
188	Guo 2015	Leukocyte homing, fate, and function are controlled by retinoic acid	Narrative / expert review	Human and preclinical (mechanistic)	2 – Human, non-ICU or mixed	3.4.3	
189	Ubesie 2013	Multiple micronutrient deficiencies among patients with intestinal failure during and after transition to enteral nutrition	Observational study	Human, non-ICU or mixed clinical settings	2 – Human, non-ICU or mixed	3.4.3; 3.4.4	Paediatric intestinal failure
190	Ross AC. Vitamin A and retinoic acid in T cell–related immunity. The American Journal of Clinical Nutrition. 2012 Nov;96(5):1166S-1172S. doi:10.3945/ajcn.112.034637 2012	Vitamin A and retinoic acid in T cell-related immunity	Narrative / expert review	Human and preclinical (mechanistic)	2 – Human, non-ICU or mixed	3.4.3	
191	Correa 2022	Vaccines, adjuvants and key factors for mucosal immune response	Narrative / expert review	Human and preclinical (mechanistic)	2 – Human, non-ICU or mixed	3.4.3	
192	Bos 2022	The role of retinoic acid in the production of immunoglobulin A	Narrative / expert review	Human, non-ICU or mixed clinical settings	2 – Human, non-ICU or mixed	3.4.3	
193	Amit-Romach 2009	Bacterial population and innate immunity-related genes in rat gastrointestinal tract are altered by vitamin A-deficient diet	Animal study	Animal model	3 – Preclinical	3.4.3	Rat model
194	Maggini 2007	Selected vitamins and trace elements support immune function by strengthening epithelial barriers and cellular and humoral immune responses	Narrative / expert review	Human, non-ICU or mixed clinical settings	2 – Human, non-ICU or mixed	3.4.4	
195	Sapkota 2018	Essential Role of Zinc and Zinc Transporters in Myeloid Cell Function and Host Defense against Infection	Narrative / expert review	Human and preclinical (mechanistic)	2 – Human, non-ICU or mixed	3.4.6	
196	von Bülow 2007	Zinc-dependent suppression of TNF-alpha production is mediated by protein kinase A-induced inhibition of Raf-1, I kappa B kinase beta, and NF-kappa B	In vitro / cell-based study	In vitro / cell-based / laboratory	3 – Preclinical	3.4.6	Cell-based
197	Chen S wen 2015	Protective effect of 1,25-dihydroxyvitamin d3 on lipopolysaccharide-induced intestinal epithelial tight junction injury in caco-2 cell monolayers	In vitro / cell-based study	In vitro / cell-based / laboratory	3 – Preclinical	3.4.6	Caco-2 monolayers
198	Heyland 2013	A randomized trial of glutamine and antioxidants in critically ill patients	Randomized controlled trial	Critically ill adults	1 – Critically ill patients	3.5; Table 4	Glutamine and antioxidants; enteral and parenteral
199	Quiroz-Olguín 2021	The effect of enteral stimulation on the immune response of the intestinal mucosa and its application in nutritional support	Narrative / expert review	Human, non-ICU or mixed clinical settings	2 – Human, non-ICU or mixed	3.5	
200	Fukatsu K. Impact of the feeding route on gut mucosal immunity: Current Opinion in Clinical Nutrition and Metabolic Care. 2014 Mar;17(2):164–70. doi:10.1097/MCO.0000000000000033 2014	Impact of the feeding route on gut mucosal immunity	Narrative / expert review	Human and preclinical (mechanistic)	2 – Human, non-ICU or mixed	3.5	

No.	First author, year	Title (as indexed)	Study type	Population / model	Evidence tier	Cited in	Note
201	MacLaren R. Considerations when administering medications enterally in the critically ill. Current Opinion in Clinical Nutrition & Metabolic Care. 2023 May;26(3):302–6. doi:10.1097/MCO.0000000000000921 2023	Considerations when administering medications enterally in the critically ill	Narrative / expert review	Critically ill adults	1 – Critically ill patients	3.5	
202	Haedge 2025	Surrogate Markers of Intestinal Permeability, Bacterial Translocation and Gut-Vascular Barrier Damage Across Stages of Cirrhosis	Observational study	Human, non-ICU or mixed clinical settings	2 – Human, non-ICU or mixed	3.6.1	Cirrhosis
203	Hardis 2022	Fecal calprotectin as a biomarker of intestinal inflammation in ICU patients with diarrhea - testing the pipette method against the collection pin …	Observational study	Critically ill adults	1 – Critically ill patients	3.6.1	Method comparison, ICU patients with diarrhoea
204	Al-Kuraishy 2022	Calprotectin: The Link Between Acute Lung Injury and Gastrointestinal Injury in Covid-19: Ban or Boon	Narrative / expert review	Human, non-ICU or mixed clinical settings	2 – Human, non-ICU or mixed	3.6.1	COVID-19
205	Faradina 2024	Micronutrient (iron, selenium, vitamin D) supplementation and the gut microbiome	Narrative / expert review	Human and preclinical (mechanistic)	2 – Human, non-ICU or mixed	3.6.2	
206	Lin 2023	The microbiome as a major function of the gastrointestinal tract and its implication in micronutrient metabolism and chronic diseases	Narrative / expert review	Human and preclinical (mechanistic)	2 – Human, non-ICU or mixed	3.6.2	

Notes. Study type and population were assigned from the publication type indexed in PubMed (where available) and from the title and abstract of each record. Entries without a PubMed record (references 109, 115, 133, 149, and 179) were classified from the reference as cited. Four records included in an earlier version of this table were removed after correction notices were identified (see Figure A1 in the manuscript) and are not listed.
